# MT1-MMP sheds LYVE-1 on lymphatic endothelial cells and suppresses VEGF-C production to inhibit lymphangiogenesis

**DOI:** 10.1038/ncomms10824

**Published:** 2016-03-01

**Authors:** Hoi Leong Xavier Wong, Guoxiang Jin, Renhai Cao, Shuo Zhang, Yihai Cao, Zhongjun Zhou

**Affiliations:** 1 Shenzhen Institute of Research and Innovation, University of Hong Kong, Shenzhen 518057, China; 2 Li Ka Shing Faculty of Medicine, School of Biomedical Sciences, University of Hong Kong, 21 Sassoon Road, Hong Kong, China; 3 Department of Microbiology and Tumor Center, Karolinska Institute, Stockholm 171 77, Sweden

## Abstract

Lymphangiogensis is involved in various pathological conditions, such as arthritis and cancer metastasis. Although many factors have been identified to stimulate lymphatic vessel growth, little is known about lymphangiogenesis inhibitors. Here we report that membrane type 1-matrix metalloproteinase (MT1-MMP) is an endogenous suppressor of lymphatic vessel growth. MT1-MMP-deficient mice exhibit spontaneous corneal lymphangiogenesis without concomitant changes in angiogenesis. Mice lacking MT1-MMP in either lymphatic endothelial cells or macrophages recapitulate corneal lymphangiogenic phenotypes observed in *Mmp14*^−/−^ mice, suggesting that the spontaneous lymphangiogenesis is both lymphatic endothelial cells autonomous and macrophage associated. Mechanistically, MT1-MMP directly cleaves LYVE-1 on lymphatic endothelial cells to inhibit LYVE-1-mediated lymphangiogenic responses. In addition, MT1-MMP-mediated PI3Kδ signalling restrains the production of VEGF-C from prolymphangiogenic macrophages through repressing the activation of NF-κB signalling. Thus, we identify MT1-MMP as an endogenous inhibitor of physiological lymphangiogenesis.

## Abstract (web summary)

Blocking lymphangiogenesis holds great therapeutic potential, however, only few inhibitors are known. Here the authors show that membrane type 1-matrix metalloproteinase suppresses lymphangiogenesis by curbing both LYVE-1-mediated proliferation of lymphatic endothelial cells and production of lymphangiogenic factors by macrophages.

## Graphical abstract

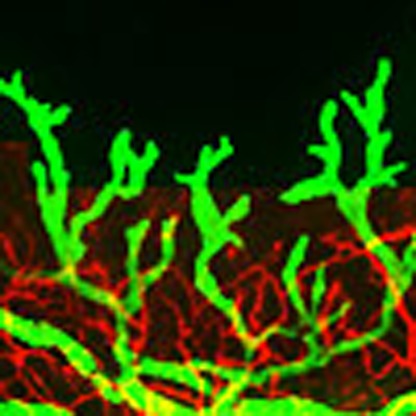

The lymphatic vasculature is required for fluid homeostasis, absorption of dietary lipids from the small intestine and trafficking of immune cells. Congenital defects in lymphatic vessel development usually result in embryonic lethality and individuals with dysfunctional lymphatic vessels often suffer from systematic oedema and impaired immune responses^1^. Lymphangiogenesis has been recently identified as a primary cause of metastasis for many cancers^1^,^2^,^3^. Despite its importance in physiology and pathological conditions, the inhibitory factors for lymphatic vessel growth remain largely unknown.

Lymphatic vessel endothelial hyaluronan receptor-1 (LYVE-1), a CD44 homologue and the predominant receptor for hyaluronan (HA) on lymphatic endothelial cell (LEC) surface, is one of the key specific markers for LECs^4^. HA is the major glyscosaminoglycan in extracellular matrix, highly enriched in connective and epithelial tissues. The binding of HA to LYVE-1 activates intracellular signalling, to promote lymphangiogenesis *in vitro*^5^.

Lymphangiogenesis during postnatal development is mainly modulated by the vascular endothelial growth factor-C (VEGF-C). *Vegf-c*^−/−^ endothelial cells can commit to the lymphatic endothelial lineages but fail to form lymphatic vessels^6^. VEGF-C and its related VEGF family member, VEGF-D, both induce lymphangiogenesis in transgenic mouse models^7^,^8^ and in tissues with exogenous recombinant VEGF-C/D^9^,^10^. VEGF-C and VEGF-D both are primary ligands for VEGF receptor 3 (VEGFR3) that is mostly expressed in the LECsduring postnatal development^11^,^12^,^13^.

Membrane type-1-matrix metalloproteinase (MT1-MMP), a membrane-boundMMP, is essential for diverse physiological and pathological processes through extracellular matrix remodelling and pericellular proteolysis^14^. Deficiency in MT1-MMP leads to many developmental defects and premature death^15^,^16^,^17^,^18^,^19^,^20^. Mutation in *MMP14* causes the multicentric osteolysis and arthritis known as Winchester syndrome^21^. As MT1-MMP-deficient mice exhibit defective fibroblast growth factor-2 (FGF2)-induced corneal angiogenesis^17^,^22^ and impaired blood vessel invasion in endochondrial ossification^17^, MT1-MMP is identified as a crucial regulator of blood vessel growth. Although the role of MT1-MMP in angiogenesis is well established, its function in lymphangiogenesis remains unexplored.

Here we show that MT1-MMP represents an endogenous negative regulator of lymphangiogenesis that acts through two pathways. It sheds cell surface LYVE-1 on LECs to restrict the lymphangiogenic potential and it is also required for maintaining PI3Kδ signalling in macrophages to inhibit their nuclear factor (NF)-κB-mediated VEGF-C production.

## Results

### Spontaneous lymphangiogenesis in *Mmp14* ^−/−^ mice

**Figure 1:**
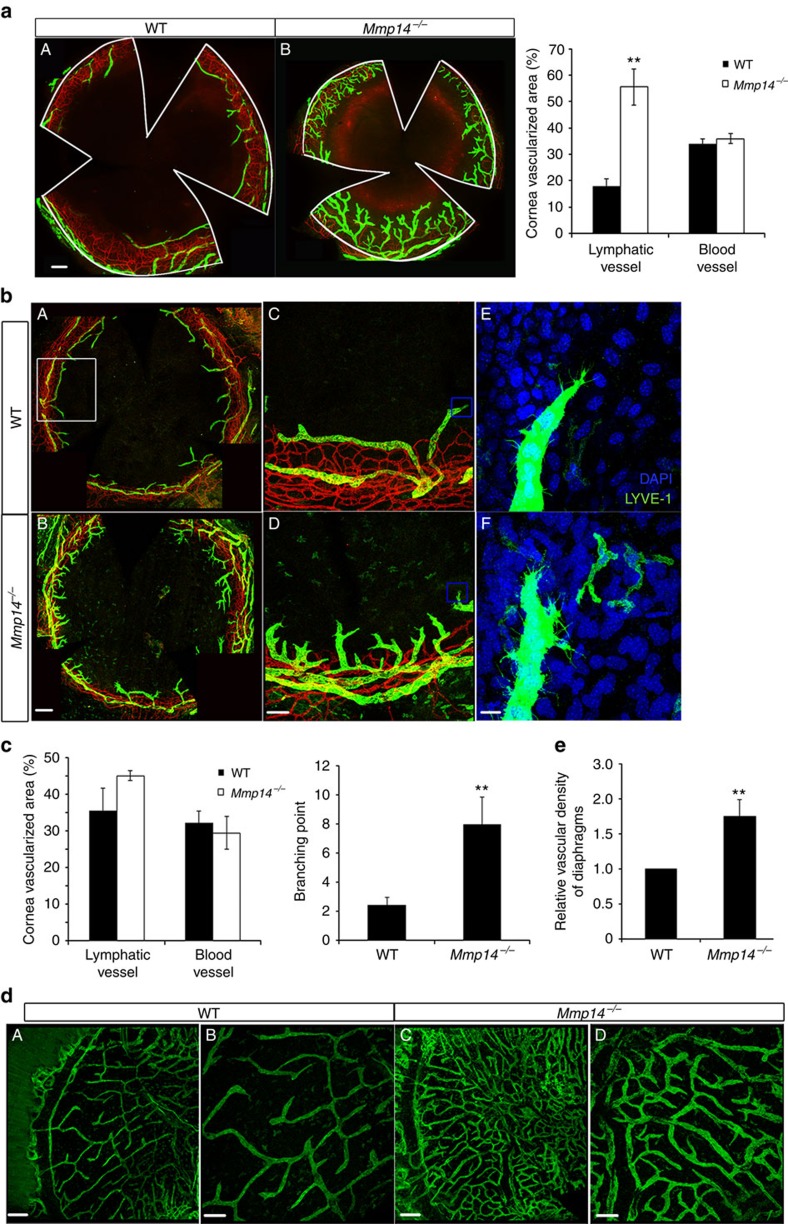
Spontaneous lymphangiogenesis in *Mmp14*^−/−^ mice. (**a**) Double immunostaining of corneal lymphatic and blood vessels using LYVE-1 (green)- and CD31(red)-specific antibodies in P18 WT (A) and *Mmp14*^−/−^ mice (B). Quantification of corneal area covered by LYVE-1^+^ lymphatic vessels and CD31^+^ blood vessels was shown in the right panel (**P<0.01, *n*=5, two-tailed *t*-test). Scale bars, 200 μm. (**b**) Corneas from P8 WT (A,C,E) and *Mmp14*^−/−^ mice (B,D,F) were immunostained with LYVE-1 (green) and CD31 (red). Images with a higher magnification in the white-boxed areas and in the yellow-boxed areas were shown in C,D and E,f, respectively. Scale bars, 200 μm (A, B), 100 μm (C,D) and 25 μm (E,F). (**c**) Quantifications of vascularization area (left panel) and branching points of each ingrowth lymphatic vessel (right panel) of **b** (**P<0.01, *n*=5, two-tailed *t*-test). (**d**) Lymphatic vessels of diaphragms from P13 WT (A,B) and *Mmp14*^−/−^ (C,D) mice were stained with LYVE-1 (green). Scale bars, 400 μm (A,C) and 200 μm(B,D). (**e**) Relative lymphatic vessel density shown in **d** (**P<0.01, *n*=5, two-tailed *t*-test). Data represent the mean±s.e.m.

To investigate the potential roles of MT1-MMP in lymphatic vessel growth, lymphangiogenesis was examined in *Mmp14*^−/−^ corneas. Flat-mounted corneas from *Mmp14*^−/−^ mice and their littermate wild-type (WT) mice were immunofluorescently stained with antibodies against LYVE-1 and CD31, two specific pan markers for LECs and vascular endothelial cells, respectively. The corneas from postnatal day 18 (P18) *Mmp14*^−/−^ mice exhibited robust spontaneous growth of lymphatic vessels without any obvious abnormality in blood vasculature (Fig. 1a). Morphometric analyses revealed significant increases in branching and area of lymphatic vessels in *Mmp14*^−/−^ corneas compared with those in WT littermates (Fig. 1a). The spontaneous corneal lymphangiogeneis could be observed as early as in P8 *Mmp14*^−/−^ mice (Fig. 1b). Although at this stage the *Mmp14*^−/−^ corneas did not exhibit significant outgrowth of lymphatic vessels from the limbus area, there were considerably more branching and sprouting of lymphatic vessels in *Mmp14*^−/−^ corneas compared with that in age-matched WT controls (Fig. 1b,c). The identity of lymphatic vessels was verified by an overlapping staining pattern between LYVE-1 and VEGFR3 (another pan marker for LECs) in corneas (Supplementary Fig. 1). These observations suggested that MT1-MMP negatively regulates lymphangiogenesis during corneal development.

To determine whether the overgrowth of lymphatic vessels in *Mmp14*^−/−^ mice is corneal specific, the lymphatic vasculature in diaphragm, another highly lymphvascularized tissue, was examined. A significant increase in lymphatic vessel density was observed in P13 *Mmp14*^−/−^ diaphragms (Fig. 1d,e), suggesting that MT1-MMP suppresses lymphatic vessel growth in non-immune privileged sites. BrdU (5-bromodeoxyuridine) pulse labelling revealed that the number of BrdU^+^ cells incorporated into the lymphatic vessels in corneas and diaphragms was approximately threefold of that in the *Mmp14*^−/−^ mice compared with that in WT mice (Supplementary Fig. 2), indicating that the spontaneous overgrowth of lymphatic vessels in *Mmp14*^−/−^ mice is probably a consequence of increased LEC proliferation.

### Elevated level of VEGF-C in *Mmp14* ^−/−^ corneas

**Figure 2:**
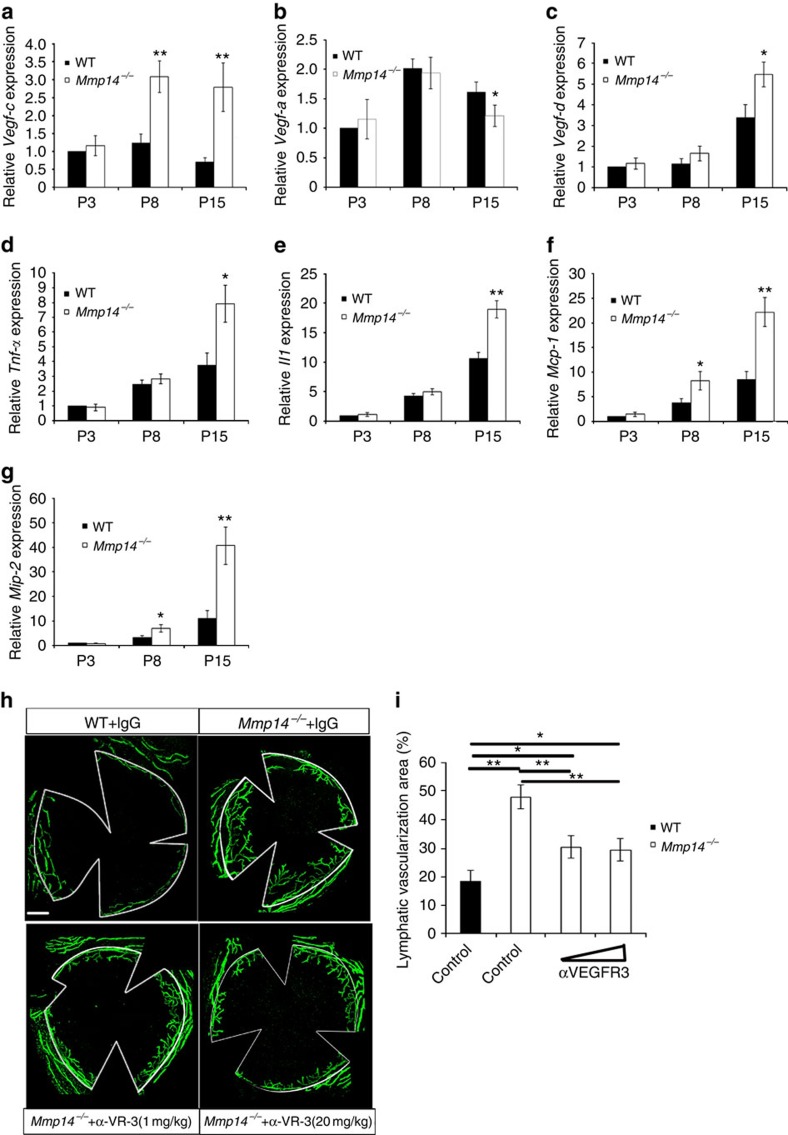
Blocking VEGFR3 activities inhibits corneal lymphangiogenesis in *Mmp14*^−/−^ mice. Real-time qPCR analyses of mRNA for *Vegf-c* (**a**), *Vegf-a* (**b**), *Vegf-d* (**c**), *Tnf-α* (**d**), *Il-1β* (**e**), *Mcp-1* (**f**) and *Mip-2* (**g**) in WT and *Mmp14*^−/−^ corneas at different ages (P3, P8 and P15) (*P<0.05,**P<0.01, *n*=5, two-tailed *t*-test). (**h**) Corneas from P15 WT and *Mmp14*^−/−^ mice with or without treatment of neutralizing antibodies against VEGFR-3 (α-VEGFR-3) were immunostained with LYVE-1 (green). Scale bars, 200 μm. (**i**) Quantification of vascularization area shown in **h** (*P<0.05; **P<0.01, *n*=5, two-tailed *t*-test). Data represent the mean±s.e.m.

As VEGFs are potent lymphangiogenic factors, we then determined and compared the transcription of different VEGFs in corneas of *Mmp14*^−/−^ mice and age-matched WT controls at different ages. The expression of *Vegf-c* was significantly upregulated in the *Mmp14*^−/−^ corneas as early as P8 (Fig. 2a). In contrast, *Vegf-a* did not change much and the upregulation of *Vegf-d* was observed in *Mmp14*^−/−^ corneas only at later stage (P15) (Fig. 2b,c). These data suggested that VEGF-C is likely to be the major factor initiating spontaneous lymphangiogenesis in *Mmp14*^−/−^ corneas. Expression of several pro-inflammatory factors including *Tnf-α*, *Il-β*, *Mcp-1* and *Mip-2* were also found elevated during corneal maturation (Fig. 2d–g). Interestingly, loss of MT1-MMP resulted in further increase in their expression, in particular at later developmental stages, indicating that MT1-MMP deficiency may have augmented the immune responses in corneas. To test whether spontaneous corneal lymphangiogenesis in *Mmp14*^−/−^ mice is a consequence of enhanced expression of VEGF-C, saturating dosage of neutralizing antibodies against extracellular domain of VEGFR3 (VEGFR3-IgG) that specifically blocks the binding of ligands to VEGFR3 was administrated intraperitoneally into *Mmp14*^−/−^ mice. The lymphatic vascularization was significantly reduced in VEGFR3-IgG-treated *Mmp14*^−/−^ corneas (Fig. 2h,i). Thus, blocking VEGFR3 largely suppresses the spontaneous lymphangiogenesis in *Mmp14*^−/−^ corneas. However, the incomplete rescue suggests that the mechanism other than VEGF-C-mediated VEGFR-3 signalling may also contribute to the increased lymphatic growth of *Mmp14*^−/−^ mice.

### LYVE-1 is a direct substrate of MT1-MMP

**Figure 3:**
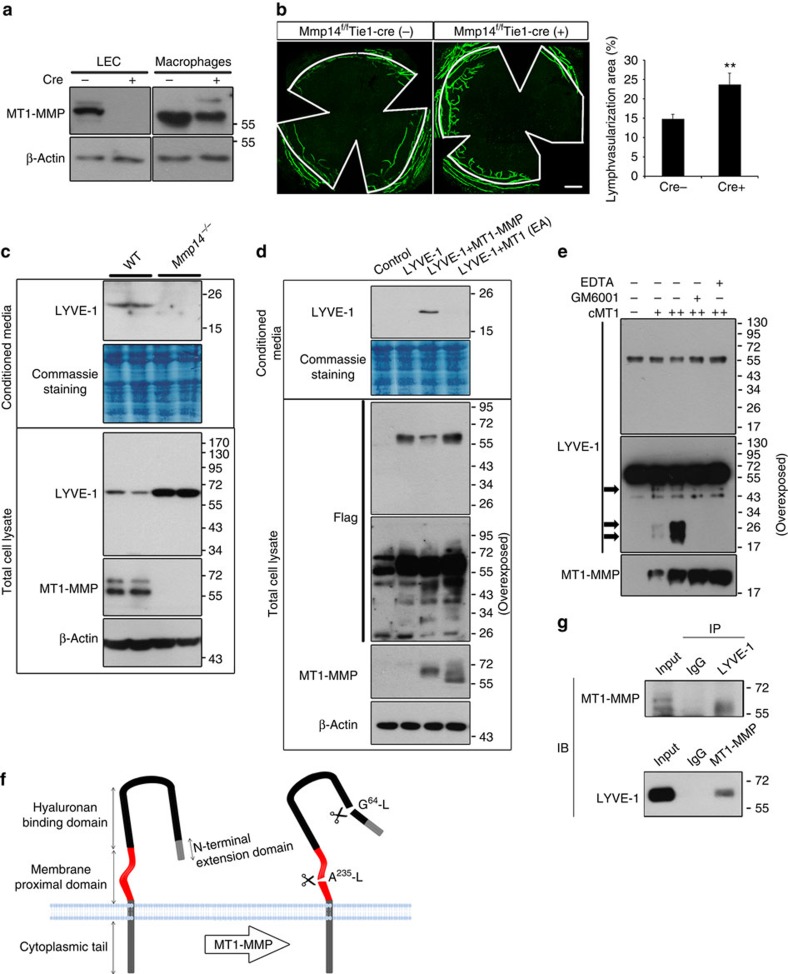
LYVE-1 is a substrate of MT1-MMP. (**a**) Western blotting analyses of MT1-MMP expression in LECs and BMMs from *Mmp14*^*f/f*^ Tie1-Cre^−^ and *Mmp14*^*f/f*^ Tie1-Cre^+^ mice. (**b**) Morphometric comparison of corneal lymphatic vascularization in *Mmp14*^*f/f*^ Tie1-Cre^−^ and *Mmp14*^*f/f*^ Tie1-Cre^+^ mice (left panel). Quantification of vascularized area was shown in the right panel (**P<0.01, *n*=5, two-tailed *t*-test). Data represent the mean±s.e.m. (**c**) MT1-MMP sheds LYVE-1 in primary LECs. The conditioned media and total cell lysates from WT and *Mmp14*^−/−^ LECs were examined by western blotting analyses using antibodies indicated. Data are representative of three independent experiments. (**d**) HEK293T cells expressing C-terminally Flag-tagged LYVE-1 were transfected with either WT or E/A catalytic mutant MT1-MMP (MT1 EA). The conditioned media and total cell lysates were subjected to western blotting analyses using antibodies indicated. Data are representative of three independent experiments. (**e**) rLYVE-1 was incubated with recombinant catalytic domain of MT1-MMP at two enzyme/substrate ratios (1:50 [+], 1:10 [++] and buffer only [−]). The protein mixture was subjected to western blotting analyses using specific antibody indicated. The cleaved fragments of LYVE-1 are indicated by black arrows. Data are representative of three independent experiments. (**f**) A diagram illustrating the predicted cleavage sites of LYVE-1 by MT1-MMP. (**g**) Endogenous interaction between LYVE-1 and MT1-MMP. LYVE-1 and MT1-MMP immunoprecipitations (IP) were generated from WT LECs using specific antibodies against LYVE-1 and MT1-MMP, and examined by western blotting analyses using indicated antibodies. IgG immunoprecipitation was used as controls. The experiments were repeated at least three times.

To explore the function of endothelial MT1-MMP in lymphangiogenesis, *Mmp14*^flox/flox^ mice were crossed with Tie1-Cre^+^ mice to generate *Mmp14*^*f/f*^ Tie1-Cre^+^ (ΔEC) mice, in which MT1-MMP was specifically depleted in endothelial cells. The expression of MT1-MMP was completely abrogated in ΔEC LECs (Fig. 3a). The lymphatic vascularization in ΔEC corneas was significantly higher than that of *Mmp14*^*f/f*^ Tie1-Cre^−^ (WT) corneas at P15 (Fig. 3b), revealing that deletion of endothelial MT1-MMP results in spontaneous corneal lymphangiogenesis. In contrast to total MT1-MMP-deficient mice, the expression of *Vegf-c* was not altered in ΔEC corneas (Supplementary Fig. 3a), suggesting that the spontaneous lymphangiogenesis resulting from the loss of endothelial MT1-MMP is independent of altered VEGF-C activities. As MT1-MMP is also expressed in the blood endothelial cells and has been found to be involved in angiogenic events^23^, we examined whether blood vessels were affected by the endothelial deletion of MT1-MMP. The morphology and the coverage of smooth muscle cells in ΔEC blood vessels in corneas were not affected (Fig. 1a,b and Supplementary Fig. 3b), suggesting the specific function of MT1-MMP in lymphatic vessels. As LYVE-1 is a specific marker for LECs and a homologue of CD44, a well-documented substrate of MT1-MMP, we examined whether the changes in lymphangiogenic responses of LECs resulting from the loss of MT1-MMP might be related to altered LYVE-1 functions. Indeed, the expression of LYVE-1 significantly increased in primary *Mmp14*^−/−^ LECs (Fig. 3c), whereas the transcription of *Lyve-1* was not altered (Supplementary Fig. 4a). Consistently, increased staining for LYVE-1 was detected in the lymphatic vessels of multiple organs in ΔEC mice (Supplementary Fig. 4b). To test whether MT1-MMP cleaves LYVE-1, either WT or activity-dead mutant MT1-MMP were transfected into HE293 cells stably expressing carboxy-terminal Flag-tagged LYVE-1. A small fragment of LYVE-1 (around 20 kDa) was detected in the conditioned media from cells transfected with WT MT1-MMP, but not in those from cells transfected with catalytic-dead MT1-MMP (Fig. 3d). Meanwhile, the full-length LYVE-1 in the total cell lysate was remarkably reduced in the total cell lysates of WT MT1-MMP-transfected cells (Fig. 3d). However, there was no additional fragment generated from the proteolytic processing of LYVE-1 by MT1-MMP (Fig. 3d longer exposure), suggesting that the cleaved fragments may subject to the rapid degradation. Consistently, the 20-kDa ectodomain fragment of LYVE-1 was also observed in the conditioned media from WT LECs but not in that from *Mmp14*^−/−^ LECs (Fig. 3c). In addition, the amount of soluble LYVE-1 was remarkably reduced in *Mmp14*^−/−^ serum, reinforcing the physiological relevance of the cleavage of LYVE-1 by MT1-MMP (Supplementary Fig. 4c). As LYVE-1 is also expressed in inflammatory macrophages^24^, we examined the MT1-MMP-mediated cleavage of LYVE-1 in peritoneal macrophages. Indeed, LYVE-1 was significantly upregulated in total cell lysates of both resting and lipopolysaccharide (LPS)-activated peritoneal macrophages isolated from irradiated WT mice transplanted with *Mmp14*^−/−^ bone marrow (Supplementary Fig. 4d). Moreover, the 20-kDa ectodomain fragment of LYVE-1 was only observed in the conditioned media of WT macrophages. These data suggest that the shedding of LYVE-1 by MT1-MMP is not LEC specific (Supplementary Fig. 4d). To further confirm the direct cleavage of LYVE1 by MT1-MMP, recombinant LYVE-1 (rLYVE-1) was incubated with the catalytic domain of MT1-MMP (cMT1) *in vitro* (Fig. 3e). Full-length rLYVE-1 was reduced and three additional fragments of LYVE-1 with molecular sizes of about 20, 30 and 50 kDa were detected in the presence of cMT1, which was inhibited by two potent MT1-MMP inhibitors, GM6001 and EDTA (Fig. 3e). The putative MT1-MMP cleavage sites of LYVE-1 were predicted to be G^64^-L and A^235^-L by Cleavpredict software^25^. To validate whether LYVE-1 is indeed cleaved by MT1-MMP at these predicted sites, two synthetic LYVE-1 polypeptides (^55^L–^75^S and ^225^E–^249^R) covering two predicted sites were digested with the recombinant catalytic domain of MT1-MMP. Mass spectrometry (MS) analyses followed by tandem MS/MS revealed that two peptides were cleaved at G^64^-L and A^235^-L, respectively (Supplementary Fig. 5). To further confirm the identified cleavage sites, the cleavage sites of LYVE-1 were mutated by site-directed mutagenesis. Glycine^64^ and alanine^235^ of LYVE-1 were mutated to alanine and valine, respectively. Mutation at G64A did not only reduce the amount of LYVE-1 fragment released into the conditioned media by MT1-MMP, but it also led to the increase in the molecular weight of the released LYVE-1 fragment by ∼10 kDa. Meanwhile, mutation at A235V and double mutations at both sites completely eliminated the released fragment of LYVE-1 in the conditioned media (Supplementary Fig. 4e). These results revealed two cleavage sites of LYVE-1 by MT1-MMP at G^64^-L within the HA-binding domain and at A^235^-L in the membrane proximal domain of LYVE-1 (Fig. 3f).

The ectodomain shedding of LYVE-1 by MT1-MMP was further substantiated by the endogenous interaction between LYVE-1 and MT1-MMP in primary WT LECs (Fig. 3g). The mature form of MT1-MMP was pulled down in the LYVE-1 immunoprecipitation. Reciprocally, LYVE-1 was detected in the MT1-MMP immunoprecipitation. The physical interaction between MT1-MMP and LYVE-1 was further confirmed in HEK293 cells ectopically expressing both LYVE-1 and MT1-MMP (Supplementary Fig. 4f).

### MT1-MMP suppresses LYVE-1-mediated lymphangiogenesis

**Figure 4:**
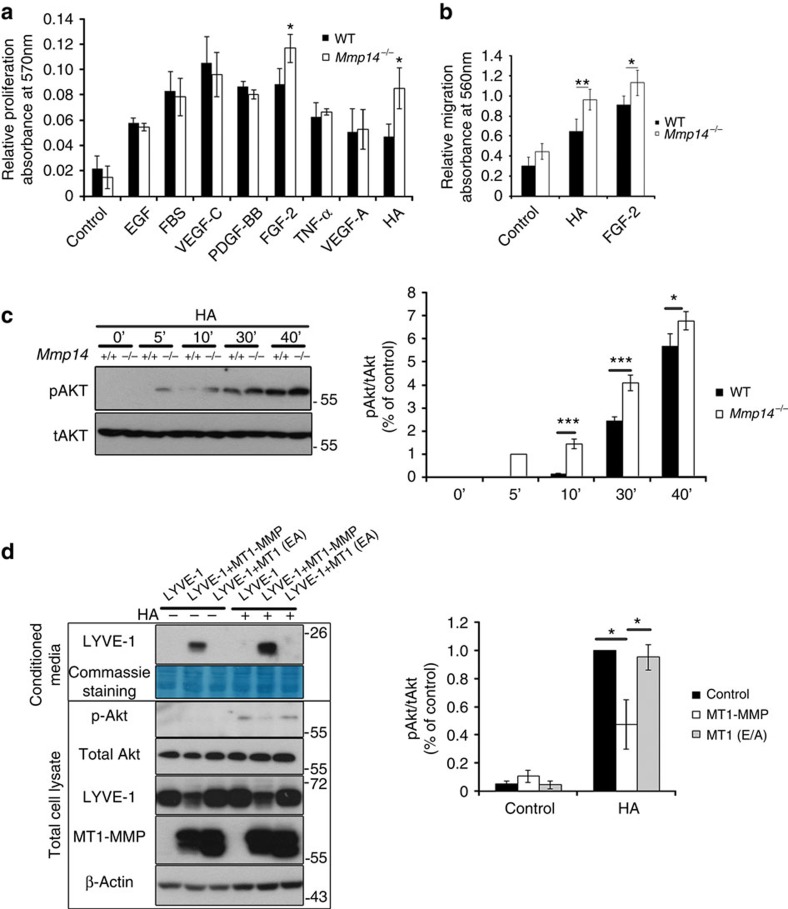
MT1-MMP is a negative regulator for LYVE-1-mediated signalling. (**a**) Serum-starved WT and *Mmp14*^−/−^ LECs were stimulated with various growth factors at (10 ng ml^−1^) and HA (3 μg ml^−1^), and subjected to MTT assays. Control cells were incubated with culture medium alone (*P<0.05, *n*=3, two-tailed *t*-test). (**b**) The cell migration of serum-starved WT and *Mmp14*^−/−^ LECs towards HA and FGF2. Control cells cultured with medium alone served as a control (*P<0.05; **P<0.01, *n*=3, two-tailed *t*-test). (**c**) Serum-starved WT and *Mmp14*^−/−^ LECs were treated with HA for indicated times. Phosphorylation of Akt was detected by western blotting (left panel). Total Akt served as a loading control. Quantification of Akt phosphorylation in relative to total Akt was shown in the right panel (*P<0.05; ***P<0.001, *n*=3, two-tailed *t*-test). (**d**) MT1-MMP sheds LYVE-1 to inhibit LYVE-1-mediated signalling. HEK293T cells stably expressing LYVE-1 were transfected with either empty vector, or WT MT1-MMP, or catalytic-dead mutant MT1-MMP (MT1 EA). The conditioned media and total cell lysates were analysed by western blotting using indicated antibodies (left panel). Phosphorylation of Akt was examined for the response of cells to HA stimulation. Quantification of Akt phosphorylation in relative to total Akt was shown in the right panel (*P<0.05, *n*=3, two-tailed *t*-test). Data represent the mean±s.e.m. The experiments were repeated at least three times.

**Figure 5:**
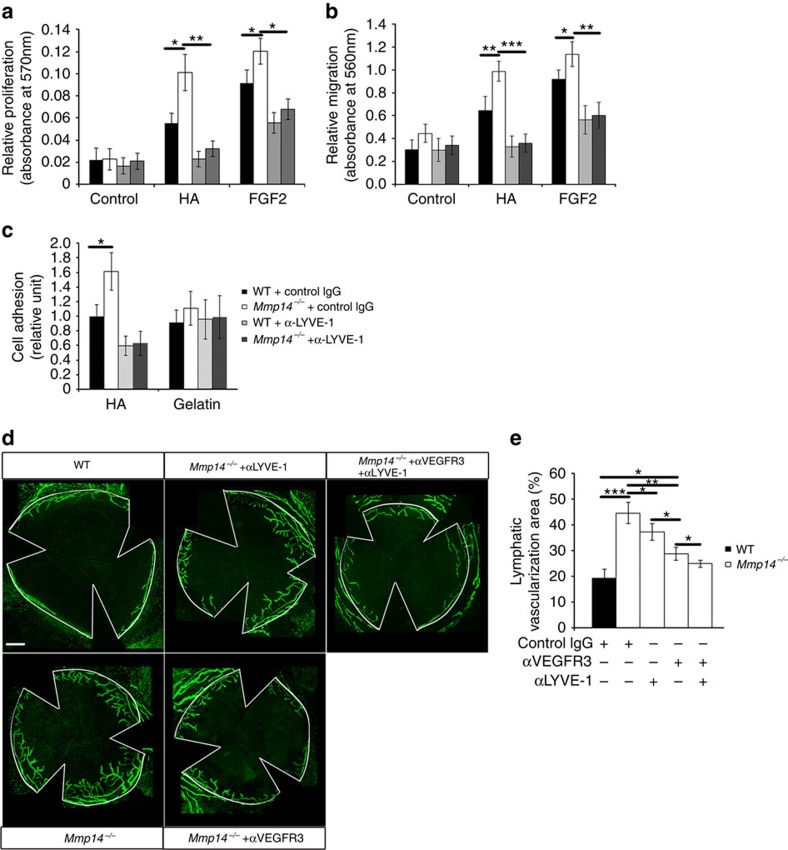
Blocking LYVE-1 suppresses corneal lymphangiogenesis in *Mmp14*^−/−^ mice. The cell proliferation (**a**) and cell migration (**b**) were examined for the responses of LECs from WT and *Mmp14*^−/−^ mice to the stimulation of either HA or FGF2. The cells were cultured either with or without the neutralizing antibody against LYVE-1 (*P<0.05; **P<0.01; ***P<0.001, *n*=3, two-tailed *t*-test). (**c**) Cell-matrix adhesion. WT and *Mmp14*^−/−^ LECs were pre-incubated with or without the neutralizing antibody against LYVE-1 and seeded in the wells coated with either HA (200 μg ml^−1^) or gelatin (50 μg ml^−1^). The adhered cells were counted and shown as relative units (*P<0.05, *n*=3, two-tailed *t*-test). (**d**) Morphometric comparison of corneal lymph vascularized areas in WT and *Mmp14*^−/−^ mice treated with or without intraperitoneal injection of neutralizing antibodies against either VEGFR3 or LYVE-1. Quantification of percentage in vascularized areas over the whole corneas was shown in **e** (*P<0.05; **P<0.01, *n*=5, two-tailed *t*-test). Scale bars, 200 μm. The statistical analyses were performed by two-tailed, unpaired Student's *t*-test. Data represent the mean±s.e.m. The experiments were repeated at least three times.

As LYVE-1 is a predominant receptor for HA on the cell surface of LECs and LYVE-1 is upregulated in *Mmp14*^−/−^ LECs, we examined whether the accumulation of LYVE-1 resulting from the loss of MT1-MMP may lead to the altered lymphangiogenic responses of LECs to HA stimulation. HA and different growth factors (for example, tumour necrosis factor (TNF)-α, platelet-derived growth factor-BB, VEGF-A/C, epidermal growth factor and FGF2) induced potent mitogenic responses in both WT and *Mmp14*^−/−^ LECs (Fig. 4a). However, *Mmp14*^−/−^ LECs proliferated faster than WT LECs in response to the stimulation of HA and FGF2 (Fig. 4a). Similarly, the migration of LECs towards HA and FGF2 was also significantly greater in *Mmp14*^−/−^ LECs than in WT cells (Fig. 4b). The increased mitogenic response to FGF2 in *Mmp14*^−/−^ LECs is consistent to a recent finding showing that LYVE-1 is essential for FGF2-induced signalling and functions in LECs^26^. Moreover, the cell adhesion onto HA, but not gelatin, was remarkably enhanced in *Mmp14*^−/−^ LECs (Fig. 5c). The HA-initiated proliferation and migration responses have been reported to be mediated by PI3k/Akt signalling in cancer cells^27^. Similarly, HA stimulated the phosphorylation of Akt in both WT and *Mmp14*^−/−^ LECs (Fig. 4c). The phosphorylation of Akt in response to HA initiated earlier and was considerably higher in *Mmp14*^−/−^ LECs (Fig. 4c). These data revealed increased abundance of functional LYVE-1 on the cell surface of LECs in the loss of MT1-MMP.

To further address whether MT1-MMP inactivates LYVE-1, HEK293 cells stably expressing LYVE-1 were transfected with either empty vector or WT or catalytic inactive MT1-MMP. Cells transfected with WT MT1-MMP exhibited decreased phosphorylation of Akt in response to the stimulation of HA (Fig. 4d), suggesting that MT1-MMP is a negative regulator for LYVE-1-mediated signalling.

To examine whether the upregulation of LYVE-1 is responsible for the augmented lymphangiogenic responses of *Mmp14*^−/−^ LECs, a neutralizing antibody against LYVE-1 was applied to specifically block the ligand binding to the cell surface LYVE-1. Indeed, the application of neutralizing antibody against LYVE-1 to the culture of *Mmp14*^−/−^ LECs effectively attenuated the increased cell proliferation and cell migration in response to the stimulation of HA and FGF2, and the enhanced cell adhesion to HA, to the levels similar to those observed in WT LECs (Fig. 5a–c). To further verify that MT1-MMP/LYVE-1 signalling axis regulates corneal lymphangiogenesis, the neutralizing antibody against LYVE-1 was administrated into the *Mmp14*^−/−^ mice. Blocking either LYVE-1 or VEGFR3 moderately but significantly suppressed the lymphatic sprouting in *Mmp14*^−/−^ corneas (Fig. 5d,e). However, blockade of VEGFR-3 is more effective than inhibiting LYVE-1 to attenuate the lymphatic growth of *Mmp14*^−/−^ corneas (Fig. 5d,e), suggesting that the inhibitory effect of MT1-MMP on lymphangiogenesis is primarily mediated through VEGFR3/VEGF-C axis. Interestingly, blocking both VEGFR3 and LYVE-1 almost completely attenuated the excessive corneal lymphangiogenesis in *Mmp14*^−/−^ mice (Fig. 5d,e). Furthermore, blocking LYVE-1, but not VEGFR3, completely inhibited the spontaneous lymphatic vessel growth in ΔEC mice (Supplementary Fig. 6),suggesting that spontaneous corneal lymphangiogenesis resulting from the deletion of endothelial MT1-MMP is primarily mediated by LYVE-1-induced angiogenic responses. To explore any functional interplay between LYVE-1-mediated lymphangiogenesis and the VEGF-C/VEGFR3 signalling pathway, we examined whether blockade of LYVE-1 affects VEGF-C-mediated functions in LECs. Blocking LYVE-1 had negligible effects on the activation of extracellular signaling regulated kinases 1/2, a well-known downstream target of VEGF-C/VEGFR3-mediated signalling (Supplementary Fig. 7a). In line with this, inhibiting LYVE-1 did not alter the VEGF-C-induced mitogenic response, whereas blocking VEGFR3 failed to change HA/LYVE-1-mediated proliferation in either WT or *Mmp14*^−/−^ LECs (Supplementary Fig. 7b), suggesting that LYVE-1 and VEGFR3 work independently in lymphangiogenesis. These results clearly demonstrated the dual roles for MT1-MMP in regulating VEGF-C/VEGFR3 signalling and LYVE-1-mediated signalling during lymphangiogenesis.

### MT1-MMP inhibits the VEGF-C production in macrophages

As the deletion of endothelial MT1-MMP did not affect the expression of VEGF-C in the cornea, the upregulation of VEGF-C on MT1-MMP loss may be attributed to other cell linages. MT1-MMP is expressed in both LECs and macrophages. Previous studies revealed that inflammatory lymphangiogenesis is dependent on CD11b^+^ macrophages recruitment and activation^28^,^29^,^30^. Macrophages are also known to be the primary source of the lymphangiogenic factors in which VEGF-C is the predominant one^29^,^30^,^31^,^32^. We therefore examined whether increased macrophage numbers in *Mmp14*^−/−^ corneas is responsible for the enhanced spontaneous lymphangiogenesis. Indeed, more CD11b^+^ macrophages were found in the central area of *Mmp14*^−/−^ corneas (Supplementary Fig. 8a,b). Prolymphangiogenic CD11b^+^ macrophages secreting VEGF-C usually express augmented level of LYVE-1 and VEGFR-3 (refs 24, 28, 33). Co-staining of CD11b together with either VEGFR3 or LYVE-1 in flat-mounted corneas revealed a remarkable increase in the number of CD11b^+^/LYVE-1^+^ and CD11b^+^/VEGFR-3^+^ macrophages in the central area of *Mmp14*^−/−^ cornea compared with that of WT littermates (Supplementary Fig. 8c,d), indicating increased numbers of prolymphangiogenic macrophages in corneas as a result of MT1-MMP deficiency. The elevated transcription of lymphangiogenic factors together with the infiltration of prolymphangiogenic macrophages provides a plausible explanation for the spontaneous lymphangiogenesis in *Mmp14*^−/−^ corneas, for example, increased VEGF-C produced from macrophages.

**Figure 6:**
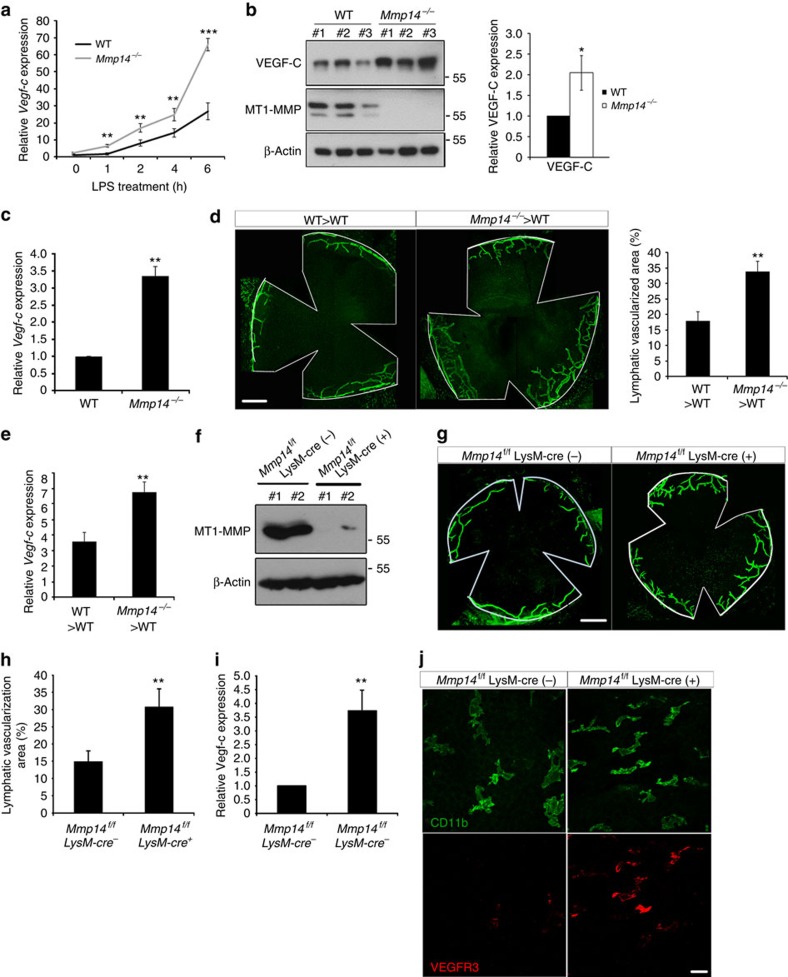
MT1-MMP restrains VEGF-C production in macrophages. (**a**) Real-time qPCR analyses of *Vegf-c* transcripts in BMMs from WT and *Mmp14*^−/−^ mice on LPS (1 μg ml^−1^) stimulation (**P<0.01, ***P<0.001, *n*=3, two-tailed *t*-test). Western blotting analyses of VEGF-C protein expression (**b**) and real-time qPCR analyses of V*egf-c* mRNA expression (**c**) in thioglycollate-elicited macrophages in WT mice transplanted with either WT or *Mmp14*^−/−^ bone marrow. Quantification of VEGF-C protein expression was shown in the right panel of **b** (*P<0.05, ***P<0.001, *n*=3, two-tailed *t*-test). (**d**) Morphometric comparison of corneal lymph vascularized areas in WT mice transplanted with either WT or *Mmp14*^−/−^ bone marrows (**P<0.01, *n*=5, two-tailed *t*-test). Scale bars, 400 μm. (**e**) qPCR analyses of *Vegf-c* mRNA levels in corneas from WT mice transplanted with either WT or *Mmp14*^−/−^ bone marrows (**P<0.01, *n*=5, two-tailed *t*-test). (**f**) Western blotting analyses of MT1-MMP in *Mmp14*^*flox/flox*^*LysM-cre*(−) and *Mmp14*^*flox/flox*^*LysM-cre* (+) peritoneal macrophages (*n*=4). (**g**) Morphometric comparison of corneal lymphvascularized areas in *Mmp14*^*flox/flox*^*LysM-cre* (−) and *Mmp14*^*flox/flox*^*LysM-cre* (+) mice at P15. The quantification of vascularization area were shown in **h** (*P<0.05, *n*=5, two-tailed *t*-test). Scale bars, 400 μm. (**i**) Real-time qPCR analyses of *Vegf-c* mRNA levels in corneas from *Mmp14*^*flox/flox*^*LysM-cre* (−) and *Mmp14*^*flox/flox*^ *LysM-cre* (+) mice at P15 (**P<0.01, *n*=3, two-tailed *t*-test). (**j**) Whole-mounted corneas from P15 *Mmp14*^*flox/flox*^*LysM-cre* (−) and *Mmp14*^*flox/flox*^ *LysM-cre* (+) mice were immunostained using VEGFR-3 (red) and CD11b (green) antibodies (*n*=5). Scale bars, 25 μm Data represent the mean±s.e.m. The experiments were repeated at least three times.

As a recent study reported that *Mmp14*^−/−^ macrophages exhibit augmented cytokine production in response to inflammatory stimuli^34^, we examined whether the loss of MT1-MMP affects VEGF-C production in response to inflammatory stimuli using bone marrow-derived macrophages (BMMs). Resting macrophages were activated with LPS, to induce VEGFR3 and LYVE-1 expressions^35^,^36^. Consistent with the previous report^34^, F4/80^+^ BMMs from *Mmp14*^−/−^ mice are morphologically indistinguishable from WT macrophages (Supplementary Fig. 9a). However, they exhibited elevated transcription of pro-inflammatory cytokines (*Il-1β* and *Il-6)* in response to LPS challenge (Supplementary Fig. 9b). In addition, BMMs from *Mmp14*^−/−^ mice showed a significant increase in the transcription of *Vegf-c* but not in *Vegf-a* or *Vegf-d* in response to LPS stimulation (Fig. 6a and Supplementary Fig. 10a). Consistently, VEGF-C protein level was also significantly elevated in *Mmp14*^−/−^ BMMs after LPS challenge (Supplementary Fig. 10b). To test whether MT1-MMP is required *in vivo* for the suppression of VEGF-C, macrophages recruited to the peritoneal cavity in response to an inflammatory stimulus were examined for VEGF-C production. Thioglycollate-elicited peritoneal macrophages were harvested from WT mice that have been irradiated and reconstituted with either *Mmp14*^−/−^ or WT bone marrow. Thioglycollate-elicited macrophages from mice reconstituted with *Mmp14*^−/−^ bone marrow expressed significantly higher levels of VEGF-C protein and messenger RNA transcript, compared with those reconstituted with WT bone marrow (Fig. 6b,c). Besides, spontaneous corneal lymphangiogenesis along with increased corneal *Vegf-c* transcription were observed in mice transplanted with *Mmp14*^−/−^ bone marrow (Fig. 6d,e). Taken together, these *in vivo* and *in vitro* results suggested that MT1-MMP suppresses VEGF-C expression in the mouse inflammatory macrophages.

### Spontaneous lymphangiogenesis is associated with macrophages

To test whether increased infiltration of macrophages contributes to the spontaneous lymphangiogenesis in *Mmp14*^−/−^ mice, we first examined the effects of LPS-activated macrophages on LECs. LPS-activated BMMs derived from either *Mmp14*^−/−^ or WT mice were co-cultured with primary LECs isolated from WT lungs. After co-culture for 24 h, LECs exhibited almost twofold increase in number when co-cultured with *Mmp14*^−/−^ BMMs in comparison with ∼60% increase when co-cultured with WT BMMs (Supplementary Fig. 11a). To test whether the prolymphangiogenic activity of LPS-activated BMMs is mainly mediated by the soluble VEGF-C, the recombinant VEGFR3/Fc protein was applied in the co-culture system to specifically trap and neutralize VEGF-C. The induction of LEC proliferation by either WT or *Mmp14*^−/−^ BMMs was largely attenuated by VEGFR3/Fc (Supplementary Fig. 11a). Similar results were observed using conditioned media derived from WT or *Mmp14*^−/−^ macrophages activated by LPS (Supplementary Fig. 11b), suggesting that the stimulating effect of macrophages on LECs is mediated largely by VEGF-C in the conditioned media. In addition, conditioned media from LPS-activated *Mmp14*^−/−^ BMMs was more potent than that of WT BMMs in promoting cell migration and tube formation of LECs. Such promoting effects were also attenuated in the presence of VEGFR3/Fc (Supplementary Fig. 11c–e). These data demonstrated that *Mmp14*^−/−^ inflammatory macrophages secrete more prolymphangiogenic VEGF-C than WT macrophages do.

To directly test the contribution of macrophages to the spontaneous lymphangiogenic phenotypes in *Mmp14*^−/−^ mice, *Mmp14*^flox/flox^ mice were crossed with LysM-Cre^+^ mice to generate *Mmp14*^*f/f*^ LysM-Cre^+^ (ΔM) mice in which MT1-MMP was specifically depleted in macrophages. ΔM mice grew normally as *Mmp14*^*f/f*^ LysM-Cre^−^ (WT) mice. The expression of MT1-MMP was almost completely abrogated in ΔM peritoneal macrophages (Fig. 6f). The area of lymphatic vascularization in ΔM corneas was significantly larger than that in WT mice (Fig. 6g,h). The corneal *Vegf-c* transcription was also remarkably elevated in ΔM mice (Fig. 6i). The corneal CD11b^+^ macrophages in ΔM mice expressed increased level of VEGFR-3 (Fig. 6j), indicating prolymphangiogenic activation of macrophages resulted from the loss of macrophage MT1-MMP. Furthermore, transplanting WT bone marrow largely attenuated the corneal lymphangiogenesis in bone marrow-depleted ΔM mice but not in ΔEC mice (Supplementary Fig. 12), indicating that the lymphatic phenotype in ΔEC mice is likely to be macrophage independent. These data demonstrated that the MT1-MMP deficiency in macrophages contributes to lymphangiogenic phenotypes in *Mmp14*^−/−^ corneas.

### MT1-MMP/PI3Kδ signalling restrains VEGF-C expression

**Figure 7:**
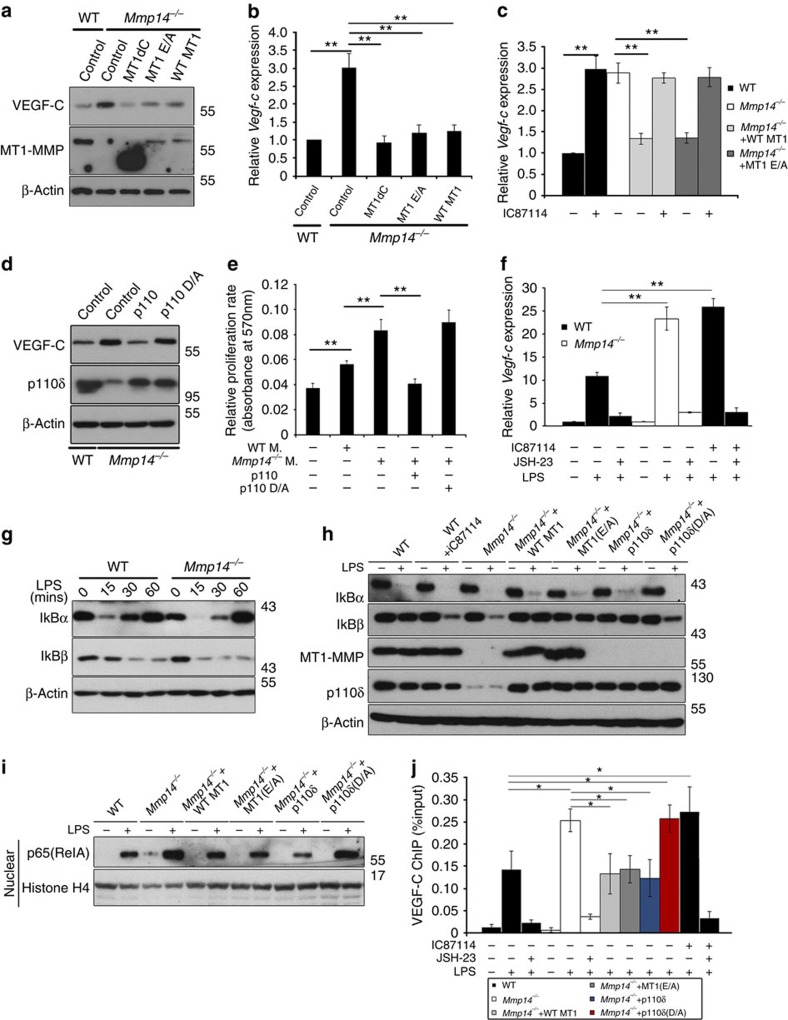
MT1-MMP suppresses VEGF-C expression via regulation of PI3Kδ signalling. (**a**) Western blot analyses of VEGF-C in WT and *Mmp14*^−/−^ BMMs with ectopic expression of control vector, full-length MT1-MMP, the cytosolic domain-deleted MT1-MMP or the E/A240 mutant MT1-MMP after stimulation with 1 μg ml^−1^ LPS for 24 h. (**b**) qPCR analyses of *Vegf-c* mRNA expression in WT and *Mmp14*^−/−^ BMMs with ectopic expression of a control vector, full-length MT1-MMP, the cytosolic domain-deleted or the E/A240 mutant MT1-MMP. Cells were stimulated with 1 μg ml^−1^ LPS for 6 h before the analyses (**P<0.01, *n*=3, two-tailed *t*-test). (**c**) qPCR analyses of *Vegf-c* mRNA in WT and *Mmp14*^−/−^ BMMs with ectopic expression of a control vector, full-length MT1-MMP or the E/A240 mutant MT1-MMP that have been incubated with or without 20 μM IC87114 for 6 h before LPS stimulation (**P<0.01, *n*=3, two-tailed *t*-test). (**d**) Western blot analyses of VEGF-C in WT and *Mmp14*^−/−^ BMMs with ectopic expression of a control vector, WT p110δ or p110δ (D910A) mutant cDNA. Cells were stimulated with LPS (1 μg ml^−1^ for 24 h) before protein analyses. (**e**) The proliferative rate of pulmonary LECs co-cultured with LPS-activated WT or *Mmp14*^−/−^ BMMs expressing a control vector, WT p110δ or p110δ (D910A) mutant cDNA measured by MTT assay (**P<0.01, *n*=3, two-tailed *t*-test). (**f**) Transcription of *Vegf-c* in WT and *Mmp14*^−/−^ BMMs by qPCR analyses. Cells were treated with or without IC87114 and JSH-23 for 4 h before LPS challenging. (**g**) Western blot analyses of IκBα and IκBβ in WT and *Mmp14*^−/−^ BMMs in response to LPS stimulation. Quantification of IκBα and IκBβ expression was shown in Supplementary Fig. 14b (*P<0.05, **P<0.01, ***P<0.001; *n*=3, two-tailed *t*-test). (**h**) Western blot analyses of IκBα and IκBβ in WT BMMs, WT BMMs treated with IC87114, *Mmp14*^−/−^ BMMs expressing a control vector or full-length MT1-MMP or E/A240 MT1-MMP, or WT p110δ cDNA or p110δ (D910A) mutant cDNA, in response to LPS stimulation for 15 min. Quantification of IκBα and IκBβ expression was shown in Supplementary Fig. 14c. (**i**) Western blotting analyses of p65 in the nuclear fractions of WT, *Mmp14*^−/−^ BMMs expressing either a control vector or full-length MT1-MMP, or E/A240 MT1-MMP, or WT p110δ or p110δ (D910A) mutant. Cells were stimulated with or without 1 μg ml^−1^ LPS for 1 h before protein extraction. Quantification of nuclear p65 was shown in Supplementary Fig. 14d (*P<0.05, *n*=3, two-tailed *t*-test). (**j**) Binding of p65 at the *Vegf-c* promoter in WT BMMs, *Mmp14*^−/−^ BMMs and *Mmp14*^−/−^ BMMs treated with a combination of LPS, IC87114 and JSH-23 were examined by qPCR of ChIP assays using a specific antibody against p65 (*P<0.05, *n*=3, two-tailed *t*-test). Data represent the average±s.e.m. The experiments were repeated at least three times.

To delineate the mechanism by which MT1-MMP suppresses the expression of VEGF-C in macrophages, *Mmp14*^−/−^ BMMs were introduced with either WT MT1-MMP or catalytically inactive MT1-MMP (MT1 E/A), or cytosolic tail-deleted MT1-MMP (MT1-MMP dC). Surprisingly, both WT and inactive mutant MT1-MMP repressed the LPS-induced expression of VEGF-C in *Mmp14*^−/−^ BMMs, to the level similar to that found in WT BMMs (Fig. 7a). Similarly, reduction in *Vegf-c* transcription was also observed (Fig. 7b). As MT1-MMP modulates inflammatory responses in macrophages independent of its catalytic activity via direct induction of PI3Kδ (p110δ) expression^34^, we speculated that MT1-MMP may regulate the expression of VEGF-C through modulation of PI3Kδ signalling. Indeed, re-introducing either WT or catalytic-inactive MT1-MMP into *Mmp14*^−/−^ BMMs restored the transcription and protein expression of PI3Kδ, to the level similar to that in WT BMMs (Supplementary Fig. 13a,b). Similar to the previous report^34^, chromatin immunoprecipitation (ChIP) with antibodies against flag in *Mmp14*^−/−^ BMMs expressing either flag-tagged WT MT1-MMP or flag-tagged mutant MT1-MMP showed that MT1-MMP could directly interact with *p110δ* promoter independent of its catalytic activity (Supplementary Fig. 13c). In macrophages, the LPS-induced PI3Kδ signalling is mainly mediated via Akt pathway^37^. LPS-induced phosphorylation of Akt was significantly compromised in *Mmp14*^−/−^ BMMs (Supplementary Fig. 13d). These results indicated that loss of MT1-MMP significantly impairs PI3Kδ/Akt signalling in BMMs. To examine the importance of PI3Kδ signalling in the regulation of VEGF-C production in BMMs, *Mmp14*^−/−^ BMMs expressing ectopic WT and enzymatic activity mutant MT1-MMP were treated with PI3Kδ-specific inhibitor, IC87114, before the LPS stimulation. IC87114 treatment enhanced LPS-induced transcription of *Vegf-c* in WT BMMs (Fig. 7c). Ectopically expressing either WT or mutant MT1-MMP in *Mmp14*^−/−^ BMMs significantly reduced the transcription of *Vegf-c*, to a level similar to that of WT BMMs. This reduction was attenuated by IC87114 (Fig. 7c), suggesting that MT1-MMP-mediated upregulation of *Vegf-c* transcription is PI3Kδ dependent. To further confirm the inhibitory function of MT1-MMP/PI3Kδ signalling on the expression of VEGF-C in inflammatory macrophages, either WT p110δ or catalytic inactive p110δ (p110δ/D910A) was ectopically expressed in *Mmp14*^−/−^ BMMs. Introduction of WT p110δ but not catalytic-inactive p110δ (p110δ/D910A) could effectively reduce the elevation of VEGF-C in *Mmp14*^−/−^ BMMs, to the level similar to that observed in WT BMMs in response to LPS stimulation (Fig. 7d). In addition, ectopic expression of WT p110δ attenuated the stimulating effect of LPS-activated *Mmp14*^−/−^ BMMs on LECs proliferation (Fig. 7e). Taken together, these results suggested that PI3Kδ signalling is involved in the regulation of VEGF-C expression in inflammatory macrophages.

Inflammatory responses elicited by extracellular stimuli such as activation of Toll-like receptor (TLR) in macrophages are usually mediated by the activation of the transcription factor NF-κB^38^. It was reported that PI3Kδ reduces inflammatory responses via suppression of nuclear translocation of NF-κB protein complex in dendritic cells^39^. Interestingly, inhibition of PI3Kδ by IC87114 promoted the nuclear translocation of NF-κB protein complex elicited by LPS in macrophages (Supplementary Fig. 14a), suggesting that the reduced PI3Kδ/AKT signalling in *Mmp14*^−/−^ macrophages may have resulted in the increased NF-κB signalling. As inflammatory induction of VEGF-C depends on NF-κB signalling^36^,^40^ and LPS-induced VEGF-C was significantly attenuated in macrophages by NF-κB-specific inhibitor, JSH-23 (Fig. 7f), it is plausible that enhanced VEGF-C production in *Mmp14*^−/−^ macrophages is a consequence of enhanced NF-κB signalling resulting from defective PI3Kδ/AKT signalling.

To test whether NF-κB signalling is involved in the regulation of VEGF-C, LPS-stimulated degradation of IκBα and IκBβ was examined, as activation of the NF-κB signalling is initiated by the degradation of IκB inhibitory proteins^41^. On LPS stimulation, *Mmp14*^−/−^ BMMs exhibited accelerated degradation of IκBα and IκBβ on LPS treatment (Fig. 7g and Supplementary Fig. 14b). In addition, nuclear translocation of the NF-κB family member p65/RelA was greatly enhanced in *Mmp14*^−/−^ BMM on LPS stimulation (Fig. 7I and Supplementary Fig. 14d). Moreover, LPS-induced binding of the *Vegf-c* promoter by RelA was significantly enriched in both *Mmp14*^−/−^ BMMs and IC87114-treated WT BMMs, examined by ChIP assay using specific RelA antibody followed by quantitative PCR (qPCR) analyses of the conserved *Vegf-c* promoter region containing a consensus Rel-A-binding motif (Fig. 7j). Ectopically expressing either WT p110δ, WT MT1-MMP or catalytic inactive MT1-MMP in *Mmp14*^−/−^ BMM completely restored the accelerated degradation of IκBα/β, the enhanced nuclear translocation of NF-κB complex and the increased binding of RelA to *Vegf-c* promoter, to the levels similar to those observed in WT BMMs (Fig. 7h–j and Supplementary Fig. 14c,d). These results suggested that loss of MT1-MMP compromises PI3kδ signalling, which in turn promotes the NF-κB nuclear translocation, leading to transcriptional activation of *Vegf-c* in inflammatory macrophages.

### Blocking p110δ activities causes corneal lymphangiogenesis

**Figure 8:**
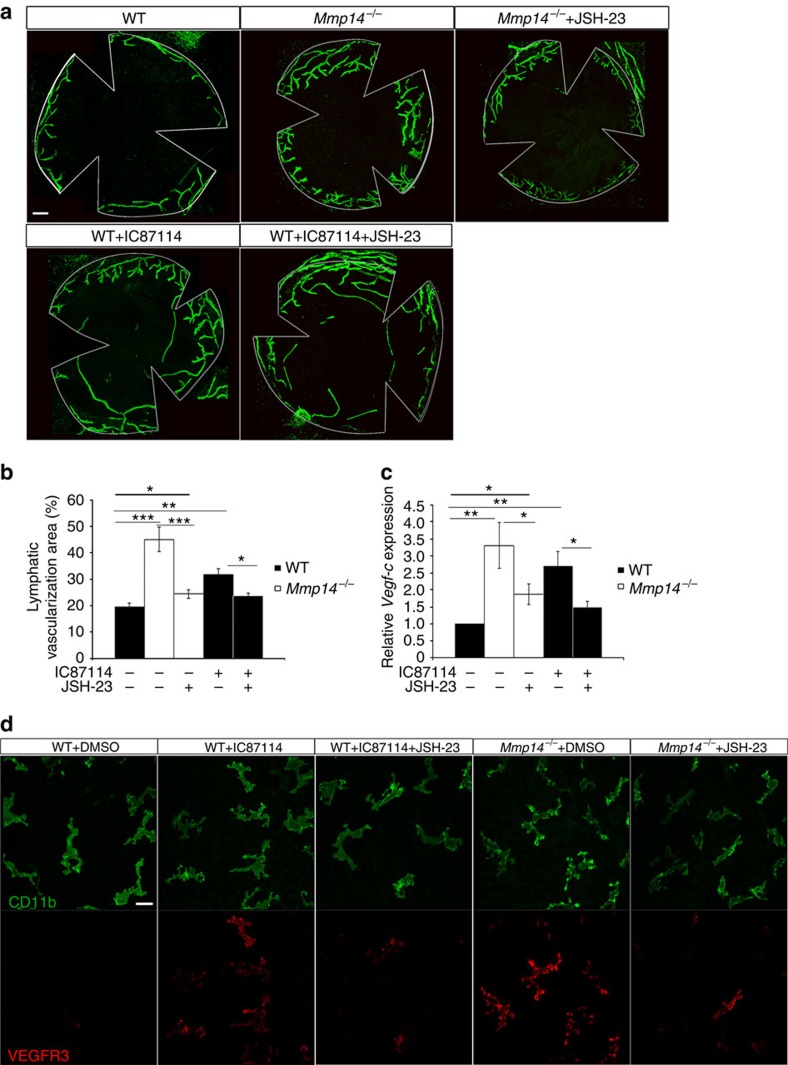
Pharmacological inhibition of P110δ leads to spontaneous corneal lymphangiogenesis. (**a**) Morphometric comparison of corneal lymph vascularized areas in WT mice treated with or without intraperitoneal injection of IC87118 (30 mg kg^−1^) and JSH23 (10 mg kg^−1^). Scale bars, 200 μm. (**b**) Quantification of percentage in vascularized areas over the whole corneas shown in **a** (***P<0.001, **P<0.01, *P<0.05, *n*=4, two-tailed *t*-test). (**c**) qPCR analyses of *Vegf-c* transcripts in corneas from WT and *Mmp14*^−/−^ mice treated with or without IC87118 (30 mg kg^−1^) and JSH23 (10 mg kg^−1^) (**P<0.01, *P<0.05, *n*=4, two-tailed *t*-test). The experiments were repeated at least three times. (**d**) Whole-mounted corneal immunostaining using VEGFR-3 (red) and CD11b (green) antibodies in P10 WT and *Mmp14*^−/−^ mice with or without the treatment of IC87118 and JSH23 (*n*=4). Scale bars, 25 μm Data represent the mean±s.e.m.

To further verify that MT1-MMP/PI3Kδ**/**NF-κB/VEGF-C axis regulates corneal lymphangiogenesis, we inhibited p110δ *in vivo* by daily intraperitoneal administration of IC87114 (30 mg kg^−1^) into WT mice. The development of lymphatic vessels in the corneas was examined 12 days later. As expected, IC87114 treatment led to increased sprouting of lymphatic vessels in corneas (Fig. 8a,b). Inhibition of NF-κB signalling *in vivo* by intraperitoneal administration of JSH-23 (10 mg kg^−1^) effectively reduced spontaneous corneal lymphangiogenesis in *Mmp14*^−/−^ mice. Importantly, JSH-23 treatment attenuated the sprouting of lymphatic vessels in WT mice treated with IC87114 (Fig. 8a,b). The spontaneous lymphangiogenesis in corneas was also associated with increased expression of *Vegf-c* (Fig. 8c) and enhanced prolymphangiogenic activation of corneal macrophages as evidenced by elevated expression of VEGFR3 in corneal CD11b^+^ macrophages in both the IC87114-treated WT mice and *Mmp14*^−/−^ mice (Fig. 8d), which could be largely inhibited by JSH-23. Furthermore, inhibition of NF-κB signalling by JSH-23 treatment, but not blockade of LYVE-1 by the treatment of LYVE-1-neutralizing antibody, completely abrogated the increased lymphatic vessel growth in ΔM corneas (Supplementary Fig. 15), suggesting that increased NF-κB signalling, but not LYVE-1-mediated signalling, is the major contributor to the lymphatic phenotype in ΔM corneas.

## Discussion

We herein showed that loss of MT1-MMP leads to spontaneous corneal lymphangiogenesis without affecting blood vasculature. These findings highlighted the importance of MT1-MMP in the control of lymphangiogenesis and the maintenance of immunological privilege in the cornea. MT1-MMP plays a dual role in the regulation of lymphangiogenesis. Although it restrains the VEGF-C production from macrophages, it also suppresses the lymphangiogenic potential of LECs. We for the first time demonstrate that LYVE-1 is a direct substrate of MT1-MMP and the MT1-MMP-mediated cleavage of LYVE-1 regulates physiological lymphangiogenesis in a VEGFR-3-independent manner. Interestingly, although LYVE-1 has long been identified as a key surface marker for lymphatic vessels, loss of LYVE-1 does not cause any obvious defects in lymphatic development^42^. Nonetheless, elevated expression of LYVE-1 may impose a profound effect on lymphangiogenesis. In fact, recent studies demonstrated similar regulatory mechanisms in other developmental processes. For instances, overexpression of CD44, the closest homologue for LYVE-1 and a well-documented substrate for MT1-MMP, has been reported to promote pathological angiogenesis and to initiate cancer progression and metastasis in various cancers, although loss of CD44 does not lead to any obvious developmental defects^43^,^44^,^45^.

This study found that MT1-MMP cleaves LYVE-1 to reduce the abundance of LYVE-1 on the cell surface of LECs. In the absence of MT1-MMP, the accumulation of LYVE-1 and higher magnitude of LYVE-1-mediated signalling promote LEC proliferation and migration, as well as LEC adhesion to the extracellular matrix, which subsequently facilitates lymphangiogenesis. However, the major factors triggering LYVE-1 activation in physiological conditions remain unclear. Degraded products of HA may be one of the major ligands initiating LYVE-1 activation, because LYVE-1 is the primary receptor for lymphatic degradation of HA^46^. FGF2 may be another possible ligand for LYVE-1 activation, as LYVE-1 is essential for FGF2-induced lymphangiogenic functions^26^. Indeed, blocking FGF2 activities with neutralizing antibody against FGF2 partially rescued the corneal lymphatic overgrowth in ΔEC mice (Supplementary Fig. 6).

MT1-MMP has a broad spectrum of substrates. In addition to LYVE-1, it may release both prolymphangiogenic and antilymphangiogenic factors from the extracellular matrix. Indeed, MT1-MMP-mediated activation of pro-MMP2 has been found to promote lymphatic outgrowth in the collagen matrix *in vitro*^47^. In contrast to these *in vitro* observations, Mmp2 deficiency leads to the increased complexity of lymphatic network *in vivo*^48^, suggesting that the *in vitro* observation may not reflect the physiological role for MMP2. Furthermore, spontaneous lymphangiogenesis resulting from MT1-MMP loss indicates that the antilymphangiogenic function of MT1-MMP probably overrides its prolymphangiogenic properties in the physiological development. Besides, among reported endogenous inhibitors of lymphangiogenesis, only mice deficient in either soluble VEGFR2 (sVEGFR2) protein or soluble sVEGFR3 generated by splicing variants of their membrane-bound forms exhibit spontaneous corneal lymphangiogenic phenotypes similar to that observed in *Mmp14*^−/−^ mice^49^,^50^. However, we failed to detect any observable alteration in their transcriptional expression in *Mmp14*^−/−^ corneas (Supplementary Fig. 16). Therefore, MT1-MMP is likely to be a sVEGFR2/sVEGFR3-independent lymphangiogenic suppressor that maintains corneal avascularity.

In addition to LECs, MT1-MMP is also expressed by macrophages that are critical in lymphangiogenesis^28^,^33^,^51^,^52^. Corneal stroma and limbus are rich in resident macrophages^53^. In the absence of MT1-MMP, these macrophages produce VEGF-C to induce spontaneous corneal lymphangiogenesis during corneal development and eyelid opening. Our study, along with previous findings^34^, demonstrated that MT1-MMP-dependent PI3Kδ signalling suppresses TLR-ligand induced NF-κB-mediated VEGF-C production in macrophages. Various endogenous TLR ligands can be found in corneas, for example, the matrix proteoglycan decorin, which is an endogenous TLR2/4 ligand enriched in the cornea and connective tissues^54^,^55^,^56^. In addition to LPS, we found that decorin also induces *Vegf-c* expression in BMMs in an NF-κB-dependent manner, which is suppressed by MT1-MMP/PI3kδ signalling (Supplementary Fig. 17). Therefore, MT1-MMP/PI3Kδ signalling may suppress inflammatory responses in macrophages induced by both endogenous and pathological TLR ligands. Although it was previously reported that MT1-MMP regulates p110δ-mediated inflammatory responses in macrophages, to the best of our knowledge it has never been shown how MT1-MMP regulates VEGF-C production. Our data, for the first time, revealed the link connecting MT1-MMP with the regulation of NF-κB-dependent VEGF-C production.

VEGF-C is not the only downstream target of NF-κB pathway. Indeed, activation of NF-κB signalling pathway induces various prolymphangiogenic inflammatory cytokines that may work synergistically in the spontaneous lymphangiogensis in *Mmp14*^−/−^ corneas. The prolymphangiogenic effect of several major NF-κB-induced factors, such as TNFα and FGF2, has been reported to be exclusively dependent of VEGFR3 signalling^9^,^57^, explaining why the VEGFR3 blockade is as effective as inhibiting NF-κB to suppress the spontaneous lymphangiogensis in *Mmp14*^−/−^ corneas.

This study revealed that MT1-MMP regulates lymphangiogenesis via two independent mechanisms. Macrophage-dependent mechanism seems to be the major contributor to the lymphatic phenotype in *Mmp14*^−/−^ corneas, as corneal lymphangiogenesis resulting from the conditional deletion of MT1-MMP in macrophages is more intensive than that in mice with MT1-MMP depletion, specifically in endothelial cells.

Increased activities of MT1-MMP have long been regarded to promote tumour metastasis^58^. However, the inhibitory effects of MT1-MMP on lymphangiogenesis suggest that targeting MT1-MMP in cancer therapy may not necessarily be beneficial, as it may promote lymphatic metastasis. Thus, it will be interesting to investigate how MT1-MMP may influence the prolymphangiogenic properties of tumour-associated macrophages, and hence tumour lymphangiogenesis and metastasis.

## Methods

### Mice

*Mmp14*^−/−^ and *Mmp14*^*flox/flox*^ mice on C57BL6 background have been previouslydescribed^17^,^19^. LyzM-cre mice were obtained from The Jackson Laboratory. Mice of both sexes were used in experiments. The age at which mice were used for experiments is shown in the figure legend. All animal experiments were performed in accordance to the guideline of the Committee on the Use of Live Animals for Teaching and Research of the University of Hong Kong.

### Antibodies

The antibodies used in this study include the following: anti-CD31 antibody (MEC13.3, 553070, BD Pharmingen; 1:150); anti-LYVE-1 antibody (11-034, AngioBio; 1:250); anti-VEGFR3 antibody (AF743, R&D System; 1:200); anti-MT1-MMP antibody (ab51074, Abcam; 1:2,000); anti-CD11b antibody (5573-94, BD Pharmingen; 1:200); an Alexa Fluor 488-conjugated anti-F4/80 antibody (MCA497A488, AbD Secrotec; 1:150); anti-BrdU antibody (Bu20a, M0744, Dako; 1:250); anti-VEGF-C (H-190, Santa Cruz, 1:1,000); anti-p65 (H-286, Santa Cruz; 1:2,000); anti-Ikβα (C-21, sc-371, Santa Cruz, 1:2,000); anti-Ikββ (S-20, sc-946, Santa Cruz, 1:2,000); anti-Akt (9272, Cell Signaling, 1:2,000); anti-p-Akt (Ser473) (9271, Cell Signaling, 1:2,000); β-actin (A1978, Sigma, 1:8,000); fluorescein isothiocyanate-conjugated goat anti-rabbit antibody (4050-02, Southern Biotech; 1:500); fluorescein isothiocyanate-conjugated donkey anti-rat antibody (6430-02, Southern Biotech; 1:500); Alexa Fluor 594-conjugated goat anti-rabbit antibody (A-11012, Invitrogen, 1:600); and Alexa Fluor 594-conjugated donkey anti-goat antibody (A11058, Invitrogen; 1:500).

### Cell isolation and culture

Primary LECs were isolated from mouse lungs. The lungs were minced and digested with 0.25% collagenase D (Roche) for 1 h at 37^o^C. Cell suspension filtered by a 70-μm cell strainer (BD Biosciences) was incubated with an anti-podoplanin (Sigma) antibody. LECs were isolated by sorting with goat anti-rabbit coated immunobeads (Miltenyi) and were cultured in PRMI-1640 supplemented with 20% fetal bovine serum (FBS), 100 μg ml^−1^ heparin (Sigma), 3 μg ml^−1^ ECGS (Sigma) and 100 ng ml^−1^ Penicillin/Streptomycin. BMMs were generated by culturing the bone marrow cells in DMEM supplemented with 10% FBS, 100 ng ml^−1^ Penicillin/Streptomycin and 10 ng ml^−1^ M-CSF (R&D) for 7 days. Peritoneal macrophages were harvested from mice that had been peritoneally injected with 2 ml of 3% Brewer thioglycollate medium for 3 days. Both types of macrophages as well as HEK 293T cells were cultured in DMEM supplemented with 10% FBS and 100 ng ml^−1^ Penicillin/Streptomycin.

### Bone marrow transplantation

Recipient WT mice at 8–10 weeks old were irradiated at 1,000 rads. Meanwhile, bone marrow cells were isolated from the femurs of WT or *Mmp14*^−/−^ mice and injected into the recipient mice via tail vein injection 4 h after irradiation. Four months after bone marrow transplantation, irradiated recipient mice reconstituted with WT or *Mmp14*^−/−^ bone marrows were killed for morphological analyses of corneal lymphatic vessel development and isolation of peritoneal macrophages. To reconstitute ΔM mice and ΔEC mice with WT bone marrow, bone marrows from male donor mice were transplanted into female recipient mice. Eight weeks after transplantation, the corneas of recipient mice were collected for morphological analyses. The transplantation efficiency was examined in accordance to ref. 59. Briefly, the bone marrow was obtained from the recipient mice at the end of transplantation experiment and the genomic DNA was isolated from the bone marrow samples (QIAamp DNA Mini kit, Qiagen). The engraftment of male donor cells in the female recipient mice was examined by measuring the expression of Y chromosome-specific gene, *Zfy1*, with real-time PCR analyses.

### Antibody and drug administration

P5 neonate mice were administered with anti-VEGFR3 antibody (mF4-31C1, ImClone) (1–20 mg kg^−1^) and anti-LYVE-1 antibody (R&D) (5 mg kg^−1^), IC87114 (30 mg kg^−1^) or JSH-23 (10 mg kg^−1^) by daily intraperitoneal injection. Injection of control IgG or dimethyl sulfoxide served as a control. On eyelid opening, drugs were applied on the corneas in eye drops. Mice were killed at P15 for phenotypic analyses. The animal care and sample analysis was not blinded to the group allocation in the animal experiments.

### DNA constructs and lentiviral transduction

Plenti6/V5-DEST plasmids expressing various mouse MT1-MMP mutants tagged with Flag at the C terminus were kindly provided by Dr Takeharu Sakamoto. The full-length mouse p110δ complementary DNA was obtained by PCR from cDNA of mouse macrophages and cloned into the plenti6/V5-DEST vector. The construct expressing full-length LYVE-1 fused with a flag tag at the C terminus was purchased from Sino Biological Inc. The construct expressing catalytic inactive p110δ were generated by mutating D910 to A. For generation of lentiviral supernatants, 293T cells cultured in DMEM supplemented with 10% FBS and 100 ng ml^−1^ Penicillin/Streptomycin were transfected with plenti6 plasmids listed above and packaging vectors (Addgene) using Lipofectamine 2000 (Invitrogen) or FuGENE HD (Roche). BMMs were infected with lentiviral supernatants in the presence of 6 μg ml^−1^ polybrene (Millipore) for 18 h, followed by drug selection with Blasticidin (3 μg ml^−1^) for 2 days. Survived cells were used for the studies.

### Cell treatment

Macrophages were pre-incubated with the PI3Kδ-specific inhibitor IC87114 (20 μM; Millipore) or the NF-κB inhibitor JSH-23 (25 μM; Sigma) for 1 h before stimulation with LPS (1 μg ml^−1^; Calbiochem) or recombinant mouse decorin (10 ng ml^−1^; R&D). Similarly, LECs were pre-incubated with the anti-LYVE-1 antibodies (R&D) (5 μM) or anti-VEGFR3 antibodies (mF4-31C1, ImClone) (10 μM) for 1 h before the stimulation with low-molecular-weighted HA (3 μg ml^−1^; Sigma). After stimulation for indicated time, cells were harvested for isolation of proteins and RNA for further analyses.

### Enzyme-linked immunosorbent assay

Mouse sera were isolated from the whole blood by allowing the blood to clot at room temperature for 30 min. The clot was removed by centrifuging the clotted blood at 2,000 *g* for 10 min at 4 °C. The concentration of LYVE-1 in serum samples was measured by enzyme-linked immunosorbent assay kit using specific antibody against mouse LYVE-1 (CSB-EL013282MO, Cusabio). Enzyme-linked immunosorbent assay was performed in accordance to the manufacturer's protocol.

### Immunohistochemistry

Whole-mount immunofluorescent staining was performed^10^. Isolated eyes and diaphragms were fixed in 4% paraformaldehyde in PBS overnight. After tissue fixation, the tissues were briefly digested with proteinase K (20 μg ml^−1^; Takara) for 5 min and blocked in 3% milk in PBST (0.3% Triton X-100 in PBS) for 16 h at 4 °C. The fixed tissues were incubated with primary antibodies overnight at 4 °C, followed by incubation with secondary antibodies for 2 h at room temperature. Immunostained positive signals of whole mounted tissues were detected by a Zeiss Confocal LSM710 microscope (Carl Zeiss). For better visualization and comparison of lymphatic vascularization among different samples, the brightness and contrast of different confocal images were adjusted to a comparable level. Meanwhile, the confocal images for comparing the expression level of target proteins were unmodified. The images were analysed by Adobe Photoshop CS5.

### Image acquisition and quantification

Image acquisition and quantification were done in accordance to the protocol^10^. Stained whole-mounted tissues were imaged by a Zeiss Confocal LSM710 microscope (Carl Zeiss) that was operated with ZEISS ZEN 2012. Low magnification (× 5 objective) was used to obtain images for the phenotypic analyses of vascularization. For detailed analyses, images were taken with higher magnifications (× 10, × 20 or × 40 objectives). A series of z-stacks images (1 μm) were captured within the z-dimensions (around 15–20 layers) containing the blood/lymphatic vessels. The microscopic images were displayed as maximum projection images. Overlapping images with low magnification were merged to obtain the image of the entire corneas by the software Adobe Photoshop CS5 (Supplementary Fig. 18a). The vascularization area refers to the percentage of corneal area covered with either lymphatic vessels or blood vessels over the total corneal area. The total corneal area was defined by outlining the innermost lymphatic vessels of the limbus (Supplementary Fig. 18b). The vascular area was outlined by the magnetic lasso tool of Adobe Photoshop (Supplementary Fig. 18c). The number of pixels in the selected vascular area was recorded and analysed by Microsoft Excel.

### *In vivo* BrdU labelling

Staining procedures were performed in accordance to the protocol^60^. Mice were intraperitoneally injected with 0.1 mg g^−1^ of BrdU solution and killed for tissue collection 3 h later. Isolated eyes and diaphragms were fixed in 4% paraformaldehyde in PBS overnight. BrdU-labelled DNA was exposed by denaturation in the formamide-SSC solution, followed by acid treatment with 2 N HCl. The remained staining procedures were performed as described above.

### Real-time PCR analyses

Total RNA was extracted from cells or tissues by TRIzol extraction (Invitrogen) and reverse transcribed into cDNA using M-MLV reverse transcriptase (Promega). qPCR was performed in StepOnePlus Real Time PCR System (Applied Biosystems) using SYBR Green PCR master mix (Takara). Gene expression was normalized with *Gapdh* mRNA levels. The sequences of gene-specific primers are shown in the Supplementary Table 1.

### ChIP assay

ChIP assay was performed using Millipore EZ-ChIP kit in accordance to the manufacturer's instructions. BMMs were fixed with 1% formaldehyde for 10 min at room temperature. The cross-linked cells were lysed in SDS lysis buffer (provided) and were sonicated in ice-cold water (six 10-s pulses; Sonics Vibra-Cell). After preclearing with Protein G-Agarose (provided) for 1 h at 4 °C, the sheared DNA was immunoprecipitated with 1 μg of anti-p65 antibody (Santa Cruz)/anti-flag antibody (Sigma) for 16 h at 4 °C and control IgG (Santa Cruz) served as a negative control. The antibody–chromatin complex was collected by Protein G-Agarose at 4 °C for 3 h. After washing the beads with washing buffers (provided) for five times, the immune complexes were eluted in elution buffer (provided). DNA was dissociated from the protein complex by incubating at 65 °C overnight. Following the digestions of RNase A and proteinase K, DNA was purified using spin columns (provided) and subjected to qPCR analyses. The specific primers for *p110δ* promoters and *Vegf-c* promoters shown in (Supplementary Table 1) were designed as previously described^34^,^40^.

### Protein extraction and western blotting

Cells were lysed in RIPA buffer (25 mM Tris-HCl, 150 mM NaCl, 1% NP-40, 1% sodium deoxycholate, 0.1% SDS, complete protease cocktail (Roche)). About 20 μg of protein lysate boiled with loading buffer was separated with SDS–PAGE electrophoresis under reducing conditions and electrotransferred onto a polyvinylidene difluoride membrane (Millipore). Following blocking with 5% (w/v) low-fat milk in PBST (0.1% Tween-20 in PBS) for 1 h at room temperature, the membrane was probed with primary antibodies diluted in blocking buffer overnight at 4 °C, followed by incubation with horseradish peroxidase-conjugated secondary antibodies for 1 h at room temperature. Western blottings were developed using enhanced chemiluminescence (ECL Plus; Pierce). The relative intensities of bands were quantified by Image J software. The original uncropped blots are shown in Supplementary Fig. 19.

### Co-immunoprecipitation

Cells were lysed in pre-chilled RIPA buffer with 300 mM NaCl. Protein mixture at the concentration of 1 μg μl^−1^ was used for the immunoprecipitation experiment. The protein lysate was immunoprecipitated with 1 μg of primary antibody at 4 °C for 16 h. The protein–antibody complex was pulled down by 50 μl Protein G-Agarose at 4 °C for 2 h. The beads were washed twice with RIPA buffer. The protein suspension was collected by boiling the beads and then subjected to western blotting analyses.

### Subcellular fractionation

Fractionation was performed as described^61^ with a few modifications. In brief, cells were lysed in pre-chilled buffer A (10 mM HEPES pH 7.9, 0.1% Triton X-100, 10 mM KCl, 1.5 mM MgCl_2_, 0.34 M sucrose, 10% glycerol, complete protease cocktail (Roche)) and incubated on ice for 5 min. Nuclei were isolated by centrifugation at 1,300 *g* for 5 min at 4 °C, followed by incubation in buffer A supplemented with 500 mM NaCl and 25% glycerol for 5 min on ice. After centrifugation at 12,000 *g* for 5 min, the supernatant was collected as the nuclear fraction that was subjected to western blotting analyses.

### Cell proliferation assay

The co-culture system consists of a transwell system with a porous membrane filter (0.4 μm pore size; Millipore) and 24-well plastic tissue culture plates. BMMs cultured in the transwell inserts were stimulated with LPS (1 μg ml^−1^) for 12 h, followed by serum starvation. Meanwhile, LECs were cultured in 24-well plates coated with 0.1% gelatin and were serum starved for 16 h on 70–80% confluence. The transwell inserts containing LPS-stimulated macrophages were placed on the LECs in 24-well plates and they were co-cultured with or without the recombinant mouse VEGFR3/Fc protein (5 μg ml^−1^; R&D) for 24 h. The culture media was removed and replaced with RPMI-1640, with 5 mg ml^−1^ MTT (3-(4,5-dimethylthiazol-2-yl)-2,5-diphenyltetrazolium bromide) for 3.5 h. The labelled LECs were solubilized in MTT solvent and the absorbance was measured at 570 nm with a reference filter of 620 nm.

### Cell migration assay

Cell migration was examined using QCM 24-Well Colorimetric Cell Migration Kit (8 μm pore size; Millipore). Briefly, conditioned media from LPS-activated BMMs was added to the lower chamber. LECs (1 × 10^5^) were seeded into the upper chamber with quenching medium (provided) in the presence or absence of VEGFR3/Fc (5 μg ml^−1^; R&D). Cells were incubated overnight and the transmigrated cells underneath the filter was stained with staining solution (provided) and solubilized with extraction buffer (provided). The absorbance was measured at 560 nm.

### Cell-matrix adhesion assay

Forty-eight-well plates were coated with various matrix substrates, such as gelatin and HA (0.2 mg ml^−1^). Serum-starved LECs were seeded into the wells at a density of 1 × 10^4^ cells per well. After the incubation for 60 min, non-adherent cells were removed by three washes of PBS. The adhered cells were counted.

### Tube formation assay

Ice-cold growth factor-reduced Matrigel (BD Biosciences) was solidified at 37 °C in 96-well plates. LECs were mixed with the conditioned media from LPS-activated BMMs with or without the addition of VEGFR3/Fc (5 μg ml^−1^; R&D) and seeded on the Matrigel. After 24 h of incubation, tube formation was visualized under bright-field inverted microscope.

### *In vitro* MT1-MMP cleavage assay

Recombinant catalytic domain of MT1-MMP (ALX-201-098-C010) and recombinant full-length LYVE-1 (H00010894-P01) were purchased from Enzo and Abnova, respectively. The rLYVE-1 consists of full-length human LYVE-1 protein (1–322 amino acids) fused with GST-tag at amino terminus. They were incubated in the assay buffer (50 mM Tris-HCl pH 7.5, 150 mM NaCl, 5 mM CaCl_2_ and 0.025% Brij35) at 37 °C for 16 h. The protein mixture was subjected to western blotting analyses.

### Mass spectrometry

The polypeptides of mouse LYVE-1 (^55^L–^75^S; ^225^E–^249^R) were synthesized at GL Biochem and incubated with recombinant catalytic domain of MT1-MMP in the assay buffer (50 mM Tris-HCl pH 7.5, 150 mM NaCl and 5 mM CaCl_2_) at 37 °C for 16 h. The reaction mixture was analysed by MS (ABI4800 MALDI TOF/TOF Analyzer). The selected peaks were further sequenced by tandem MS/MS.

### Statistical analyses

Each experiment was independently repeated at least three times. Tissues from at least three independent and randomly chosen mice at comparable developmental stages were collected for analyses and none of the samples was excluded from analyses. The sample size was increased in accordance to the statistical variation. Data are represented as mean±s.e.m. The variances from multiple groups of independent samples were analysed by F-test. The statistical analysis was performed by two-tailed, unpaired Student's *t*-test. All data meet the normal distribution. *P*-value <0.05 was considered statistically significant.

## Additional information

**How to cite this article:** Wong, H. L. X. *et al*. MT1-MMP sheds LYVE-1 on lymphatic endothelial cells and suppresses VEGF-C production to inhibit lymphangiogenesis. *Nat. Commun.* 7:10824 doi: 10.1038/ncomms10824 (2016).

## Supplementary Material

Supplementary Information Supplementary Figures 1-19 and Supplementary Table 1
