# Supplementary material for: MT1-MMP sheds LYVE-1 on lymphatic endothelial cells and suppresses VEGF-C production to inhibit lymphangiogenesis
Source: Nat Commun. 2016 Mar 1;7:10824. doi: 10.1038/ncomms10824 (PMC4773521; doi:10.1038/ncomms10824)
Supplement: Supplementary Information — Supplementary Figures 1-19 and Supplementary Table 1 [file ncomms10824-s1.pdf]

Supplementary Figure 1

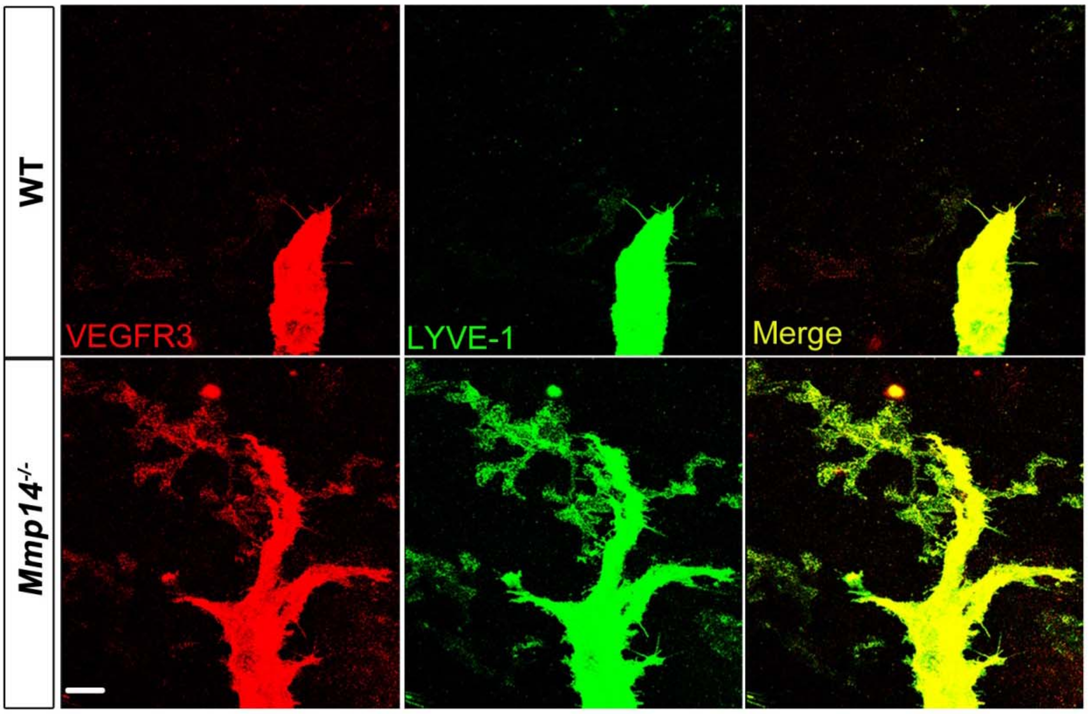

Supplementary Figure 1. Verification of lymphatic vessel identity in corneas

Double immunostaining of corneal lymphatic vessels using VEGFR-3 (red) and LYVE-1 (green) antibodies in wild-type and *Mmp14*$^{-/-}$ mice. Scale bars: 50 μm

Supplementary Figure 2

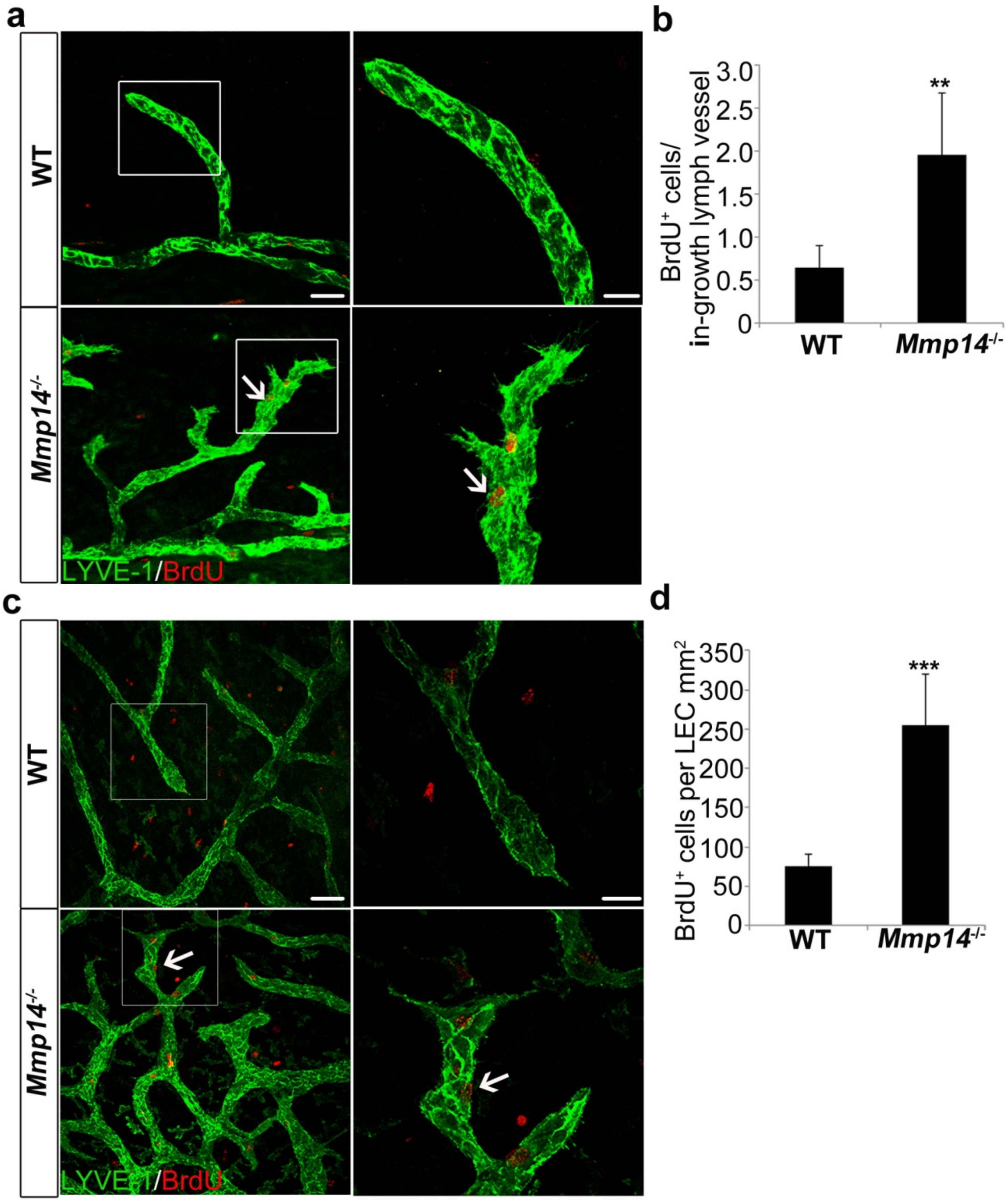

Supplementary Figure 2. Increased LECs proliferation in *Mmp14$^{-/-}$* corneas and diaphragms

(a) Double immunostaining of corneas from P10 wild-type and *Mmp14$^{-/-}$* mice using BrdU (red) and LYVE-1 (green) specific antibodies. The images with higher magnification in the white boxed areas were shown in the right panel. BrdU positive cells incorporated into ingrowth lymphatic vessels are indicated by white arrows. Scale bars: 100µm (left panel); 25µm (right panel)

(b) Quantification of BrdU positive cells shown in **(a)** (**$p<0.01$; n=5, two-tailed *t*-test)**.**

(c) Diaphragms from P13 wild-type and *Mmp14$^{-/-}$* mice double-stained with BrdU (red) and LYVE-1 (green). BrdU positive cells are indicated by white arrows. Scale bars: 100µm (left panel); 25µm (right panel)

(d) Quantification of BrdU positive cells shown in **(c)** (***$p<0.001$; n=5, two-tailed *t*-test). Data represent the average ± SEM.

Supplementary Figure 3

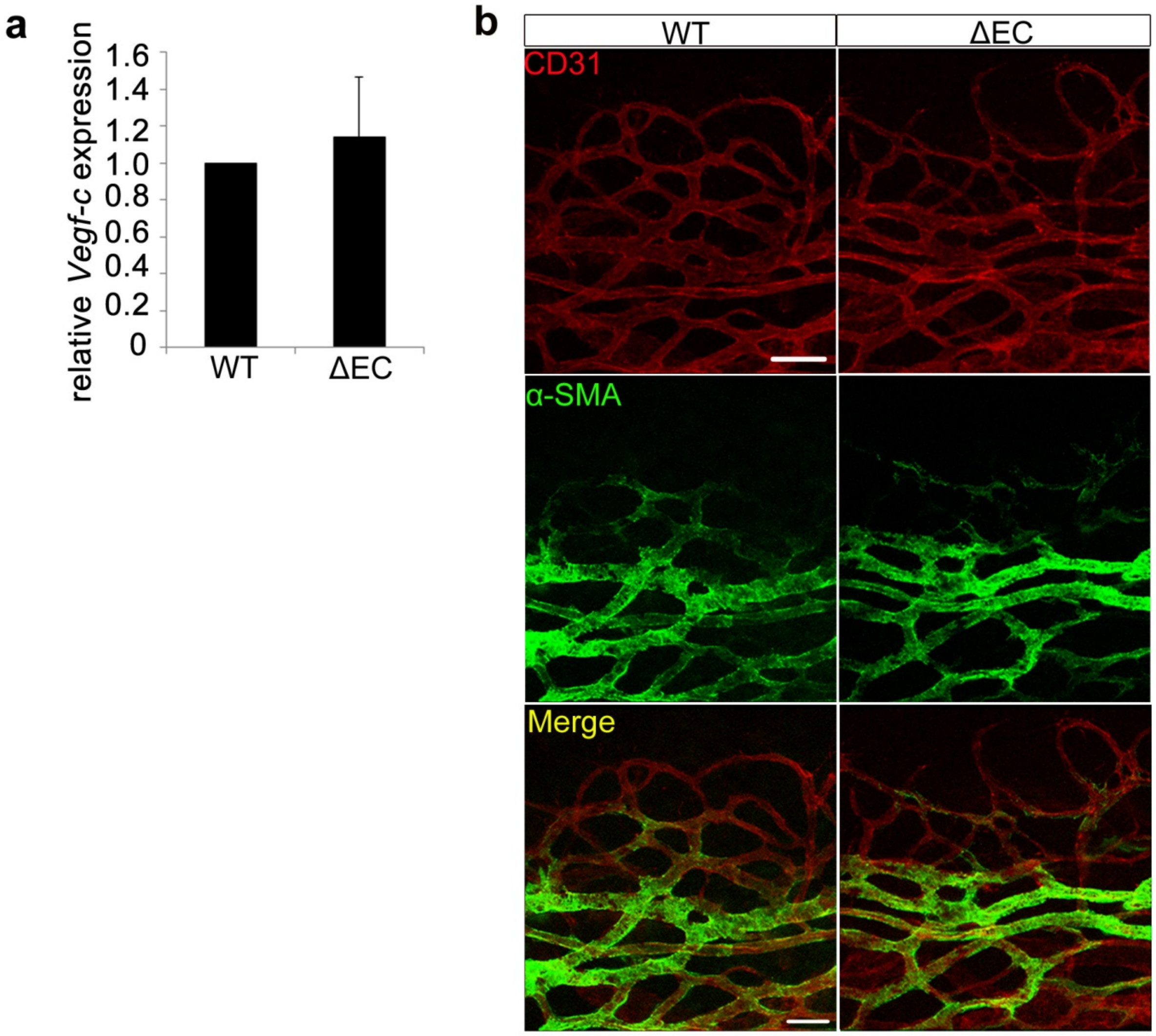

Supplementary Figure 3. Endothelial deletion of MT1-MMP does not affect the morphology of blood vessels

(a) Real time qPCR analyses of *Vegf-c* mRNA levels in corneas from *Mmp14*$^{flox/flox}$*Tie1-cre (-)* (WT) and *Mmp14*$^{flox/flox}$ *Tie1-cre (+)* (ΔEC) mice at P15. Data represent the average ± SEM; n=4

(b) Morphological analysis of smooth muscle coverage in blood vessels. Corneas from P15 WT and ΔEC mice were doubly stained with specific antibodies against CD31 (red) and α-smooth muscle actin (α-SMA, green). Scale bars: 100 μm

Supplementary Figure 4

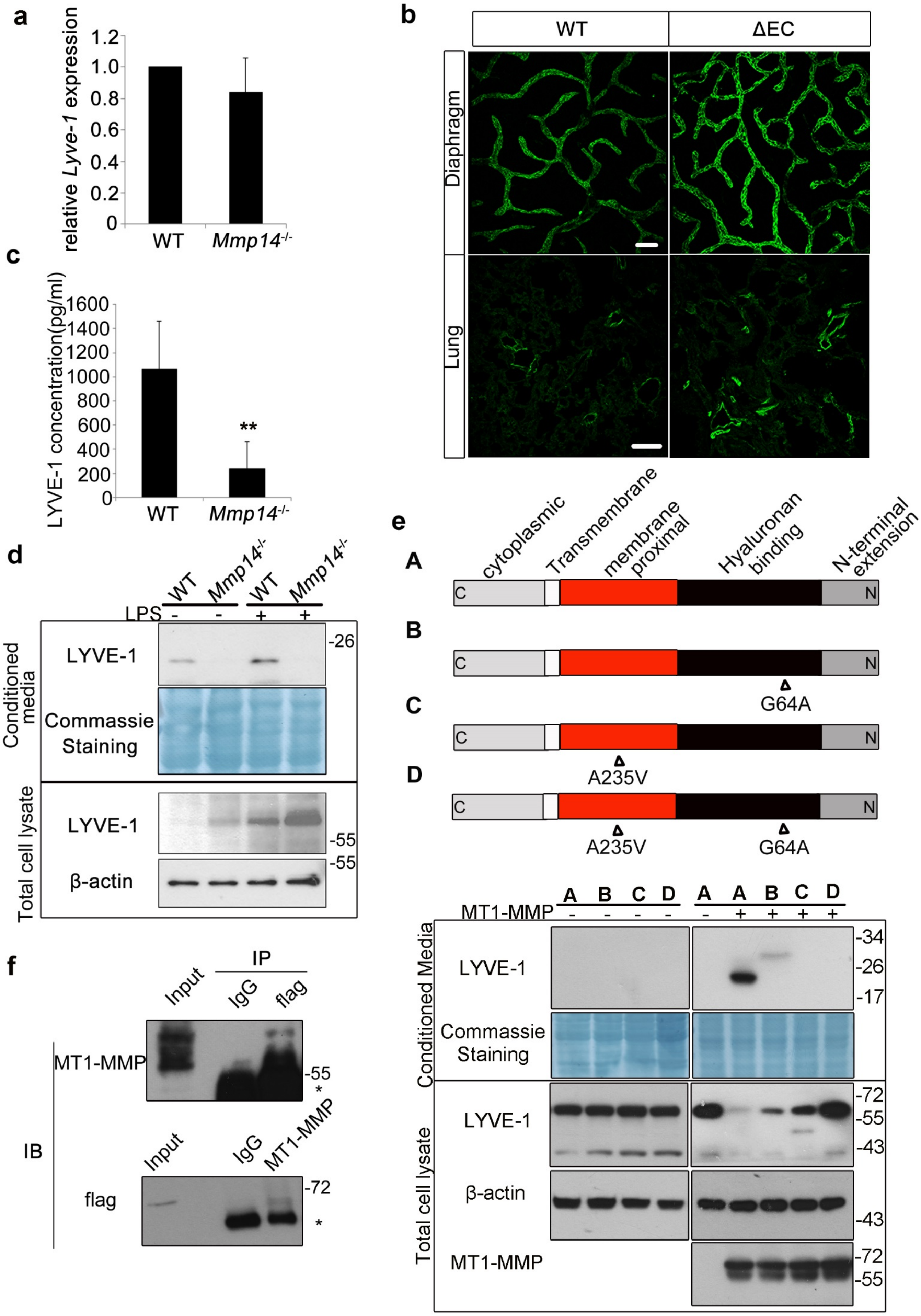

Supplementary Figure 4 MT1-MMP cleaves LYVE-1 to inhibit LYVE-1 mediated signaling

(a) Real time qPCR analyses of *Lyve-1* mRNA levels in lymphatic endothelial cells from wild-type (WT) or *Mmp14*$^{-/-}$ mice.

(b) The expression of LYVE-1 was examined by the immunofluorescence staining of whole-mount diaphragms and sections of lungs from *Mmp14*$^{flox/flox}$Tie1-cre (-) (WT) and *Mmp14*$^{flox/flox}$ Tie1-cre (+) (ΔEC) mice at P15. Scale bars: 400μm (diaphragms) 100μm (lung)

(c) The concentrations of LYVE-1 in sera from P15 wild-type and *Mmp14*$^{-/-}$ mice were quantified by ELISA using specific antibody against LYVE-1. (**p<0.01; n=4, two-tailed *t*-test)

(d) Western blotting analyses on the expression of LYVE-1 in thioglycollate-elicited macrophages in wild-type mice transplanted with either wild-type or *Mmp14*$^{-/-}$ bone marrow. These macrophages were stimulated with or without 1 μg/ml LPS for 16 hrs prior to the analyses.

(e) Mutations of predicted cleavages sites block the cleavage of LYVE-1 by MT1-MMP. Cartoon illustrations of the mutated sites in LYVE-1: (A) wild-type LYVE-1 (B) LYVE-1 with G64A mutation (C) LYVE-1 with A235V mutation (D) LYVE-1 with double mutations at G64A and A235V. HEK293T cells expressing different mutated LYVE-1s (A-D) were transfected with either empty vector or wild-type MT1-MMP. The conditioned media and total cell lysates were analyzed by Western blotting using indicated antibodies (lower panel).

(f) Co-immunoprecipitation of ectopic MT1-MMP and LYVE-1 in HEK293T cells. LYVE-1 and MT1-MMP immunoprecipitations (IP) were prepared from the lysates of HEK293T cells ectopically expressing MT1-MMP and Flag-tagged LYVE-1 using antibodies against MT1-MMP and flag, respectively. The IP samples were subjected to western blotting analyses using indicated antibodies. Control IgG was used as control. * indicates the IgG heavy chain.

Data represent the average ± SEM. The experiments were repeated at least three times.

Supplementary Figure 5

a

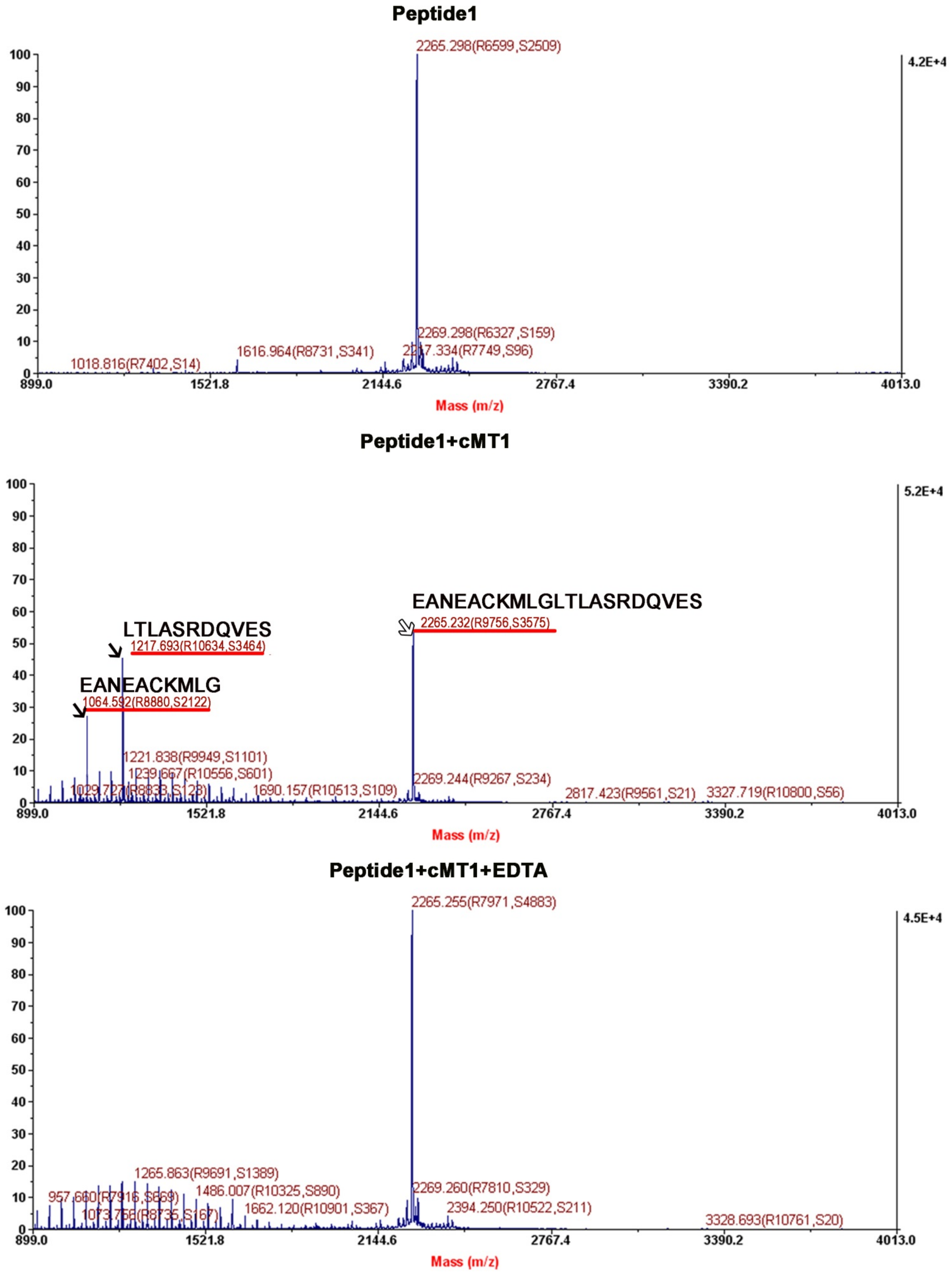

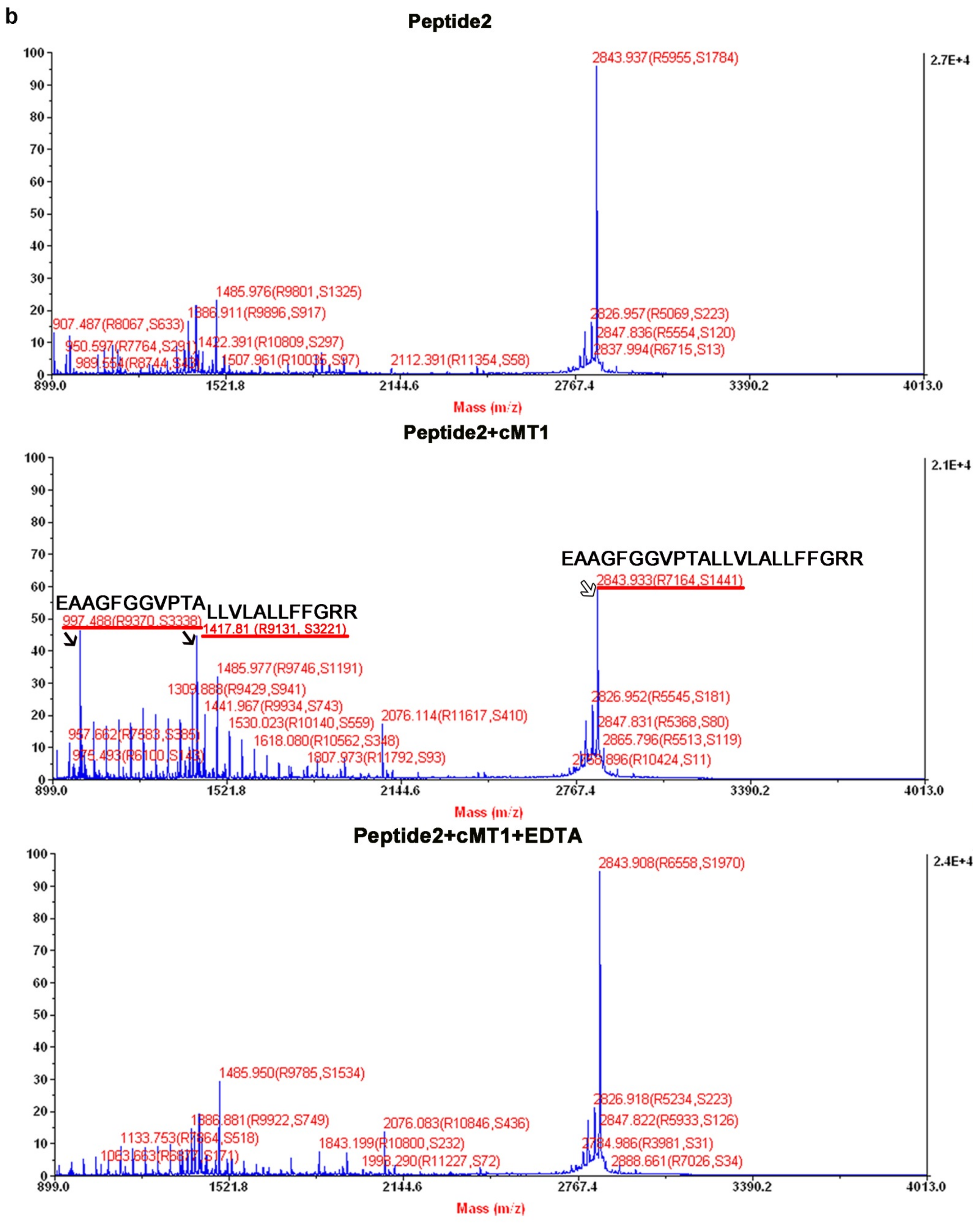

Supplementary Figure 5 Recombinant MT1-MMP cleaves LYVE-1 polypeptides. (a-b) Mass spectrometry analyses after incubating two LYVE-1 polypeptides [**(a)** ^{55}L-^{75}S and **(b)** ^{225}E-^{249}R] with recombinant catalytic domain of MT1-MMP. The control experiments were performed in the presence of EDTA inhibition. **(a)** The intact peptide 1 with molecular weight of 2265.2 Da was cleaved into two

small fragments in the presence of recombinant MT1-MMP. The fragments at 1064.6 Da and at 1217.7 Da were further identified as EANEACKMLG and LTLASRDQVES, respectively, by tandem MS/MS. **(b)** The peptide 2 at 2843.9 Da was broken in two smaller fragments. The fragments at 997.5 Da and at 1417.8 Da were further sequenced as EAAGFGGVPTA and LLVLALLFFGRR, respectively, by tandem MS/MS.

Supplementary Figure 6

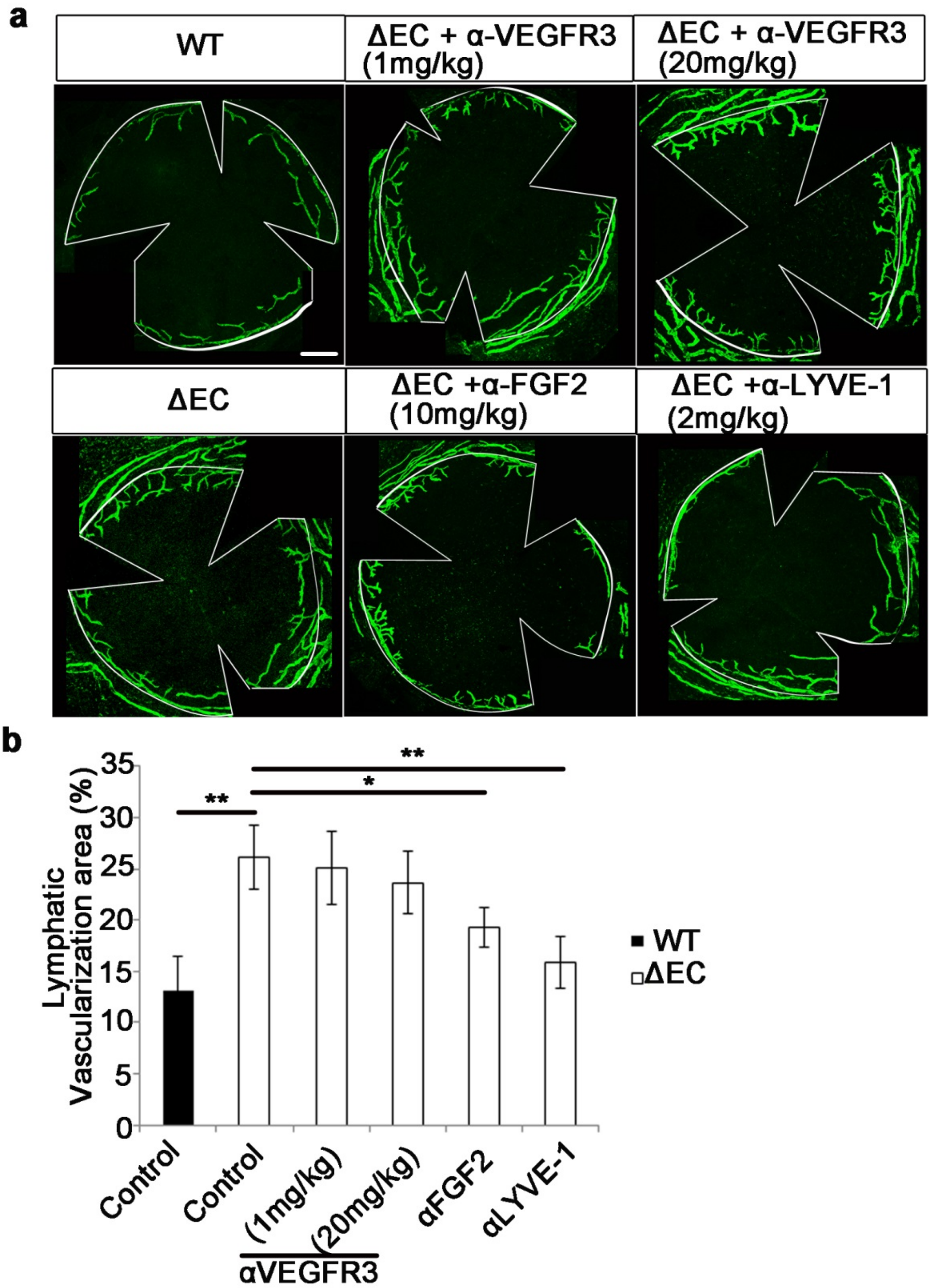

Supplementary Figure 6 Endothelial MT1-MMP regulates LYVE-1-mediated angiogenic responses *in vivo*

(a) P5 *Mmp14*$^{f/f}$ Tie1-Cre^{-} (WT) and *Mmp14*$^{f/f}$ Tie1-Cre^{+} (ΔEC) mice were treated daily with either α-VEGFR3 neutralizing antibody (1 or 20 mg/kg), α-FGF2 neutralizing antibody (10 mg/kg) or α-LYVE-1 neutralizing antibody (2 mg/kg) via intraperitoneal injection. Mice treated with control IgG served as controls. Mice were sacrificed at P20 for the morphological examination of lymphatic vascularization in corneas. Corneas were immunostained with LYVE-1 (green). The quantification of lymphatic vascularization was shown in **(b).** The statistical analyses were performed by ANOVA, followed by two-tailed, unpaired Student's t test. Data represent the average ± SEM. (**p*<0.05; ***p*<0.01, n=4-5) Scale bars: 200μm

Supplementary Figure 7

a

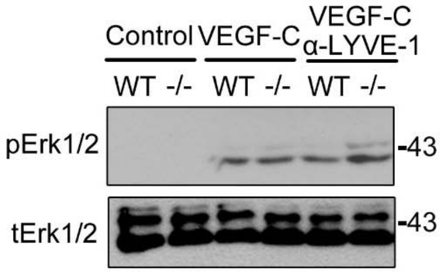

b

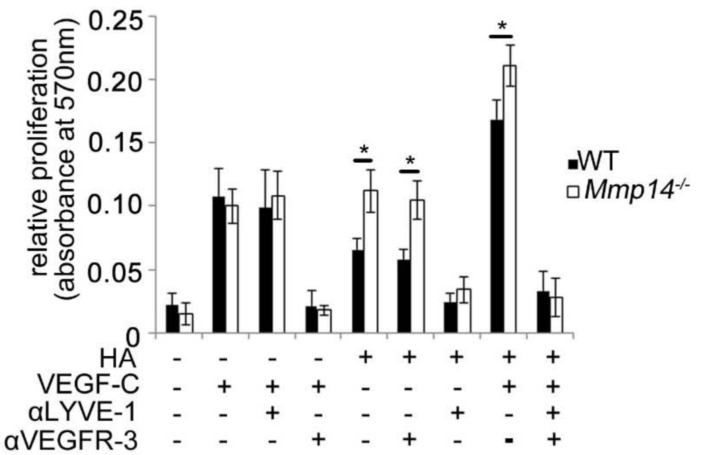

Supplementary Figure 7 VEGF-C mediated proliferative responses in LECs are independent of LYVE-1

(a) Serum-starved wild-type (WT) and *Mmp14*$^{-/-}$ LECs were treated with either HA or HA plus neutralizing antibodies against LYVE-1. The phosphorylation of Erk was examined by western blotting using indicated antibodies. tErk served as a loading control.

(b) Serum-starved wild-type (WT) and *Mmp14*$^{-/-}$ LECs were treated with either HA, or VEGF-C, or the neutralizing antibody against LYVE-1, or the neutralizing antibody against VEGFR-3 or their combined treatments. The cell proliferation in response to the treatments was measured by MTT assay. (*p<0.05; n=3, two-tailed t-test) Data represent the average ± SEM. The experiments were repeated at least three times.

Supplementary Figure 8

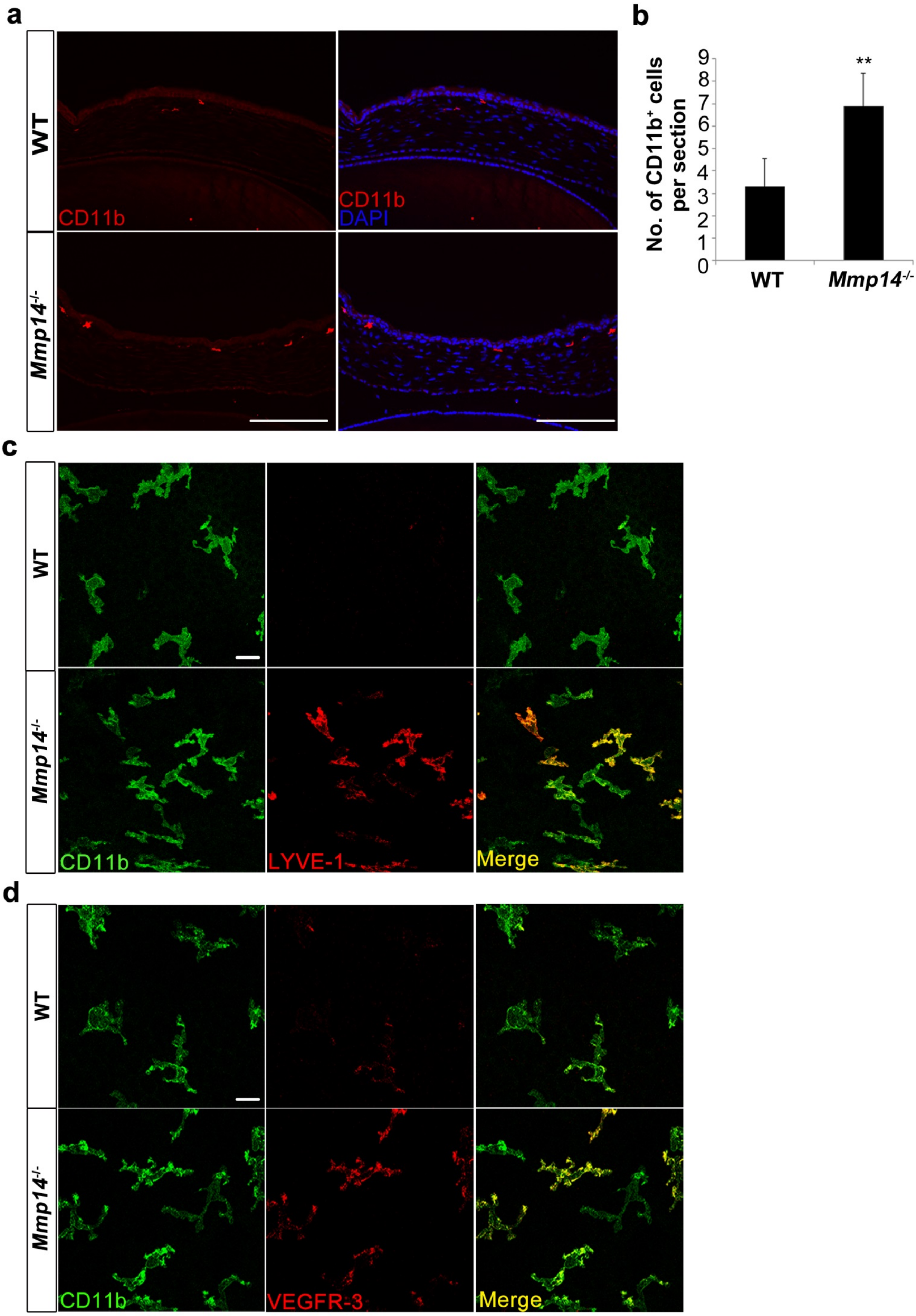

Supplementary Figure 8. Infiltration of $CD11b^{+}$ activated macrophages in *Mmp14*$^{-/-}$ corneas

(a) Corneal section of P8 wild-type and *Mmp14*$^{-/-}$ mice were immunostained using CD11b (red) antibody. Nuclear staining was visualized by DAPI (blue). Scale bars: 100 µm

(b) Quantification of $CD11b^{+}$ macrophages per section of corneas shown in **a** (***p*<0.01, n=5, two-tailed *t*-test). Data represent the average ± SEM.

(c) Whole mounted corneas from P8 wild-type and *Mmp14*$^{-/-}$ mice were immunostained using LYVE-1 (red) and CD11b (green) antibodies. Scale bars: 25 µm

(d) Whole mounted corneas from P8 wild-type and *Mmp14*$^{-/-}$ mice were immunostained using VEGFR-3 (red) and CD11b (green) antibodies. Scale bars: 25 µm

Supplementary Figure 9

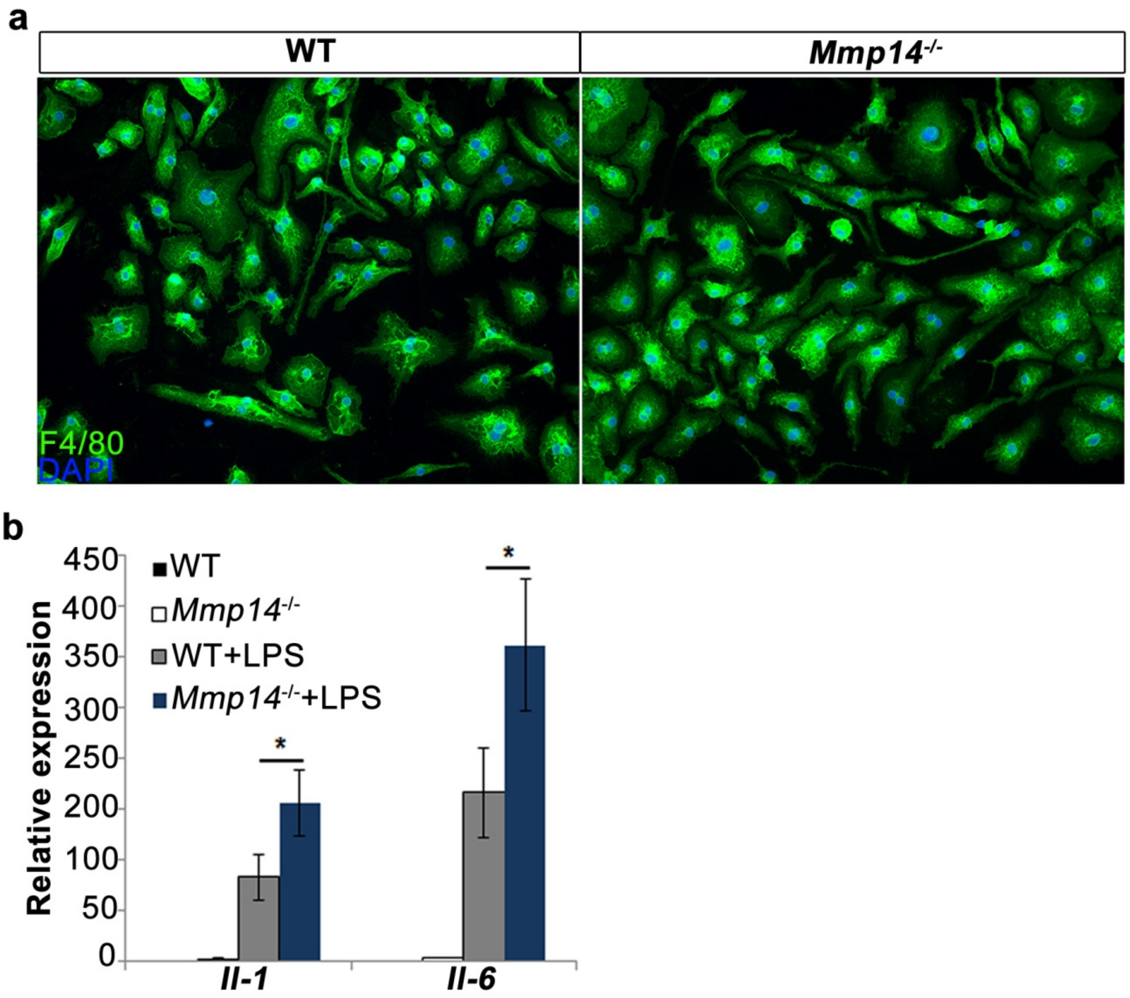

Supplementary Figure 9. Comparison of morphology and cytokine production between wild-type and *Mmp14*$^{-/-}$ bone marrow-derived macrophages

(a) BMMs from wild-type and *Mmp14*$^{-/-}$ mice were immunostained with macrophage marker, F4/80 (green). Nuclear staining was shown by DAPI (blue).

(b) qPCR analyses of *Il-1β and Il-6* mRNA expressions in wild-type and *Mmp14*$^{-/-}$ BMMs with or without LPS treatment (**p*<0.05; n=3, two-tailed *t*-test). Data represent the average ± SEM. The experiments were repeated at least three times.

Supplementary Figure 10

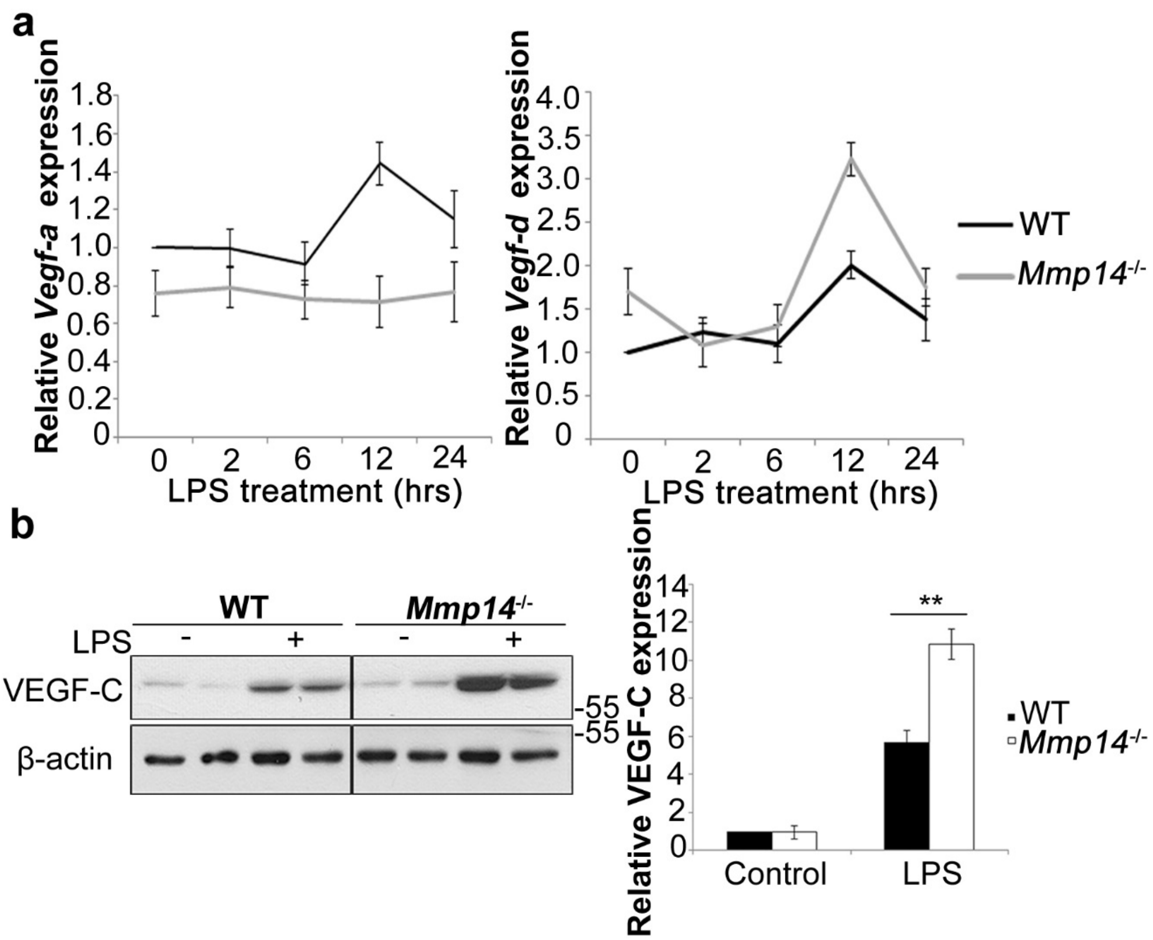

Supplementary Figure 10. Deregulated expression of VEGFs in *Mmp14*$^{-/-}$ macrophages

(a) qPCR analyses of *Vegf-a & Vegf-d* mRNA levels in BMMs from WT mice and *Mmp14*$^{-/-}$ mice upon LPS-stimulation (1μg/mL).

(b) Western blotting analyses of VEGF-C expression in BMMs from WT mice and *Mmp14*$^{-/-}$ mice upon LPS-stimulation (1μg/mL for 24 hours). β-actin served as a loading control. (**p<0.01; n=3, two-tailed t-test). Data represent the average ± SEM. The experiments were repeated at least three times.

Supplementary Figure 11

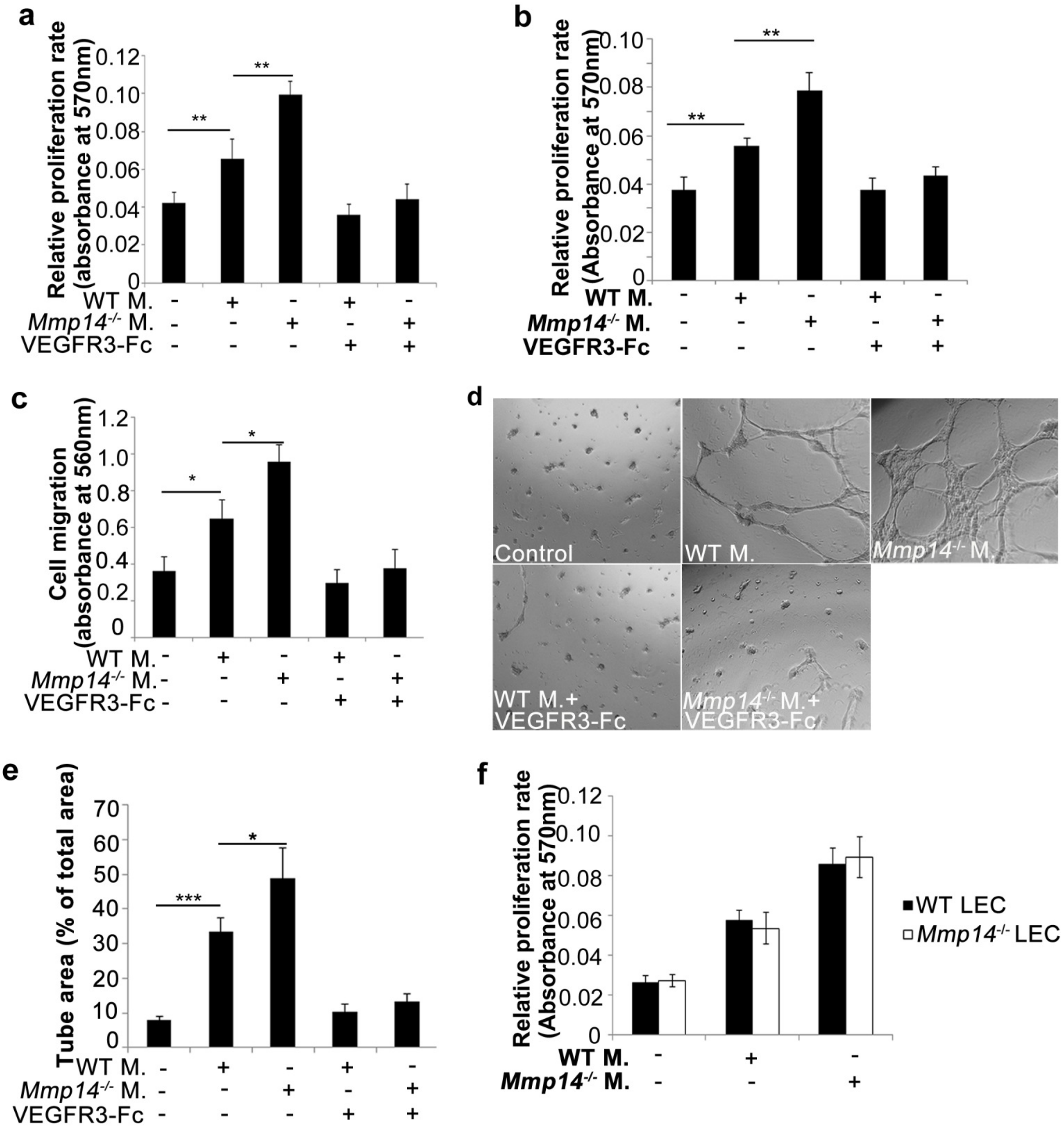

Supplementary Figure 11. Activated *Mmp14*$^{-/-}$ macrophages are more pro-lymphangiogenic than wild-type macrophages

(a) Proliferation of primary pulmonary LECs co-cultured with LPS-activated wild-type or *Mmp14*$^{-/-}$ BMMs measured by MTT assay (***p*<0.01; n=4, two-tailed *t*-test).

(b) Proliferation measured by MTT assay in primary pulmonary LECs co-cultured with conditioned media from LPS activated macrophages derived from wild-type or *Mmp14*$^{-/-}$ bone marrow derived macrophages (***p*<0.01, n=4, two-tailed *t*-test)

(c) Migration of LECs cultured with conditioned media from LPS-activated wild-type or *Mmp14*$^{-/-}$ BMMs examined by transwell migratory assay (**p*<0.05; n=4, two-tailed *t*-test).

(d) Tube forming potential of LECs cultured with conditioned media from LPS-activated wild-type or

Mmp14$^{-/-}$ BMMs assessed by *in-vitro* tube formation assay. Quantification of percentage of area covered by LEC tubule structure was shown in **(e)** (*$p<0.05$; *** $p<0.001$; n=4, two-tailed *t*-test).

(f) Proliferation measured by MTT assay (n=4) in primary pulmonary LECs derived from WT or *Mmp14*$^{-/-}$ mice that have been co-cultured with conditioned media from LPS activated macrophages derived wild-type or *Mmp14*$^{-/-}$ bone marrow. Data represent the average ± SEM. The experiments were repeated at least three times.

Supplementary Figure 12

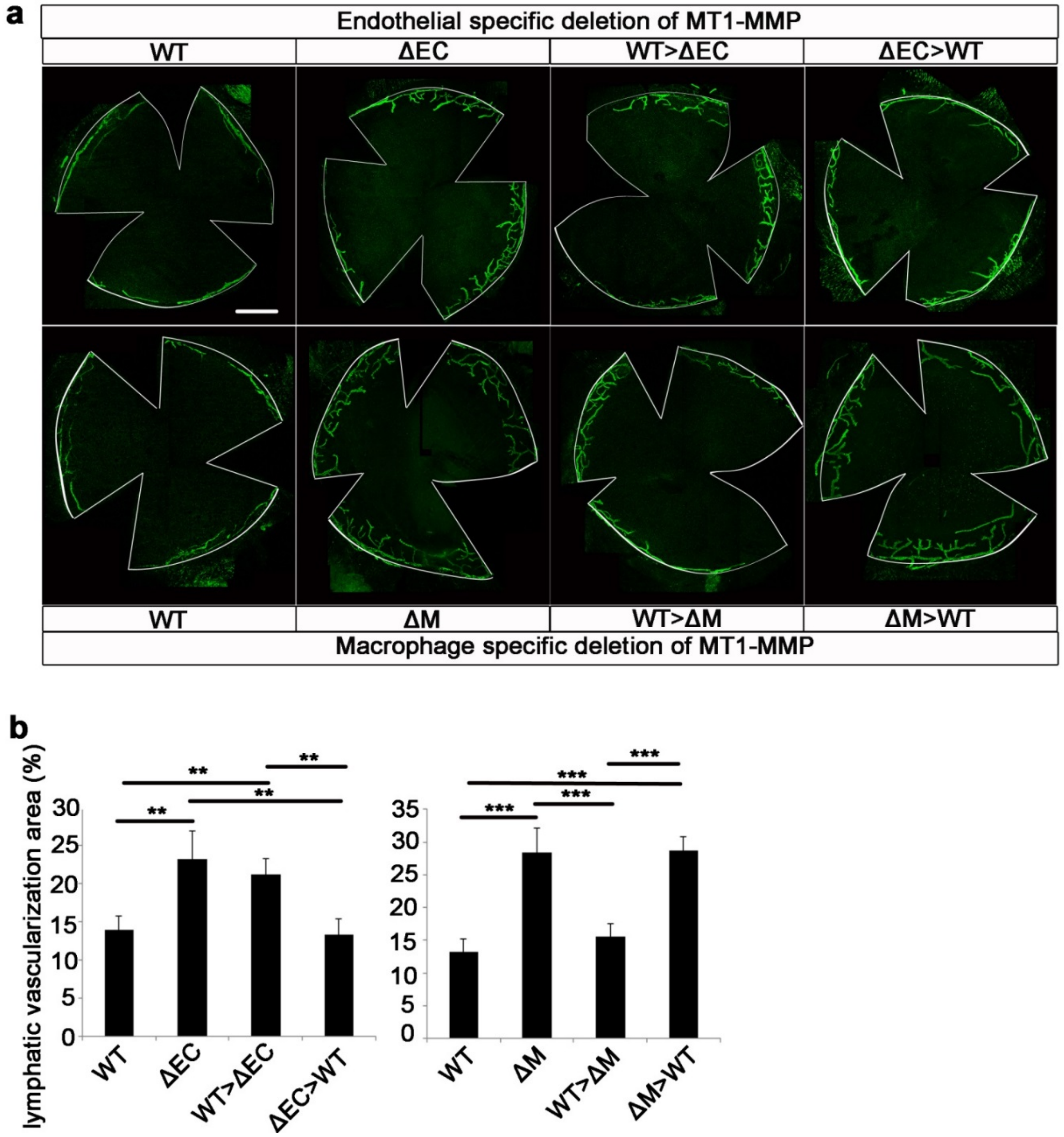

Supplementary Figure 12. The spontaneous corneal lymphangiogenesis resulted from the loss of endothelial MT1-MMP is not associated with macrophages

(a) Morphometric comparison of corneal lymphvascularized areas in *Mmp14*$^{flox/flox}$*Tie1-cre* (-)/ *Mmp14*$^{flox/flox}$*LysM-cre* (-) mice (WT), *Mmp14*$^{flox/flox}$*Tie1-cre* (+) mice (ΔEC mice), *Mmp14*$^{flox/flox}$*LysM-cre* (+) mice (ΔM), irradiated ΔEC mice transplanted with *Mmp14*$^{flox/flox}$*Tie1-cre* (-) bone marrow (WT>ΔEC), irradiated ΔM mice transplanted with *Mmp14*$^{flox/flox}$*LysM-cre* (-) mice (WT>ΔM) and irradiated wild-type mice transplanted with either ΔEC or ΔM bone marrows (ΔM>WT; ΔEC>WT). Corneal lymphatic vessels in ΔM>WT and ΔEC>WT mice were analysed 8 weeks after bone marrow transplantation by immunostaining with LYVE-1 (green). Quantification of lymphatic vascularization area was shown in **(b)**. The statistical analyses were performed by ANOVA, followed by two-tailed, unpaired Student's t test. Data represent the average ± SEM. The experiments were repeated at least three times. (**$p<0.01$; ***$p<0.001$, n=4-5) Scale bars: 200μm

Supplementary Figure 13

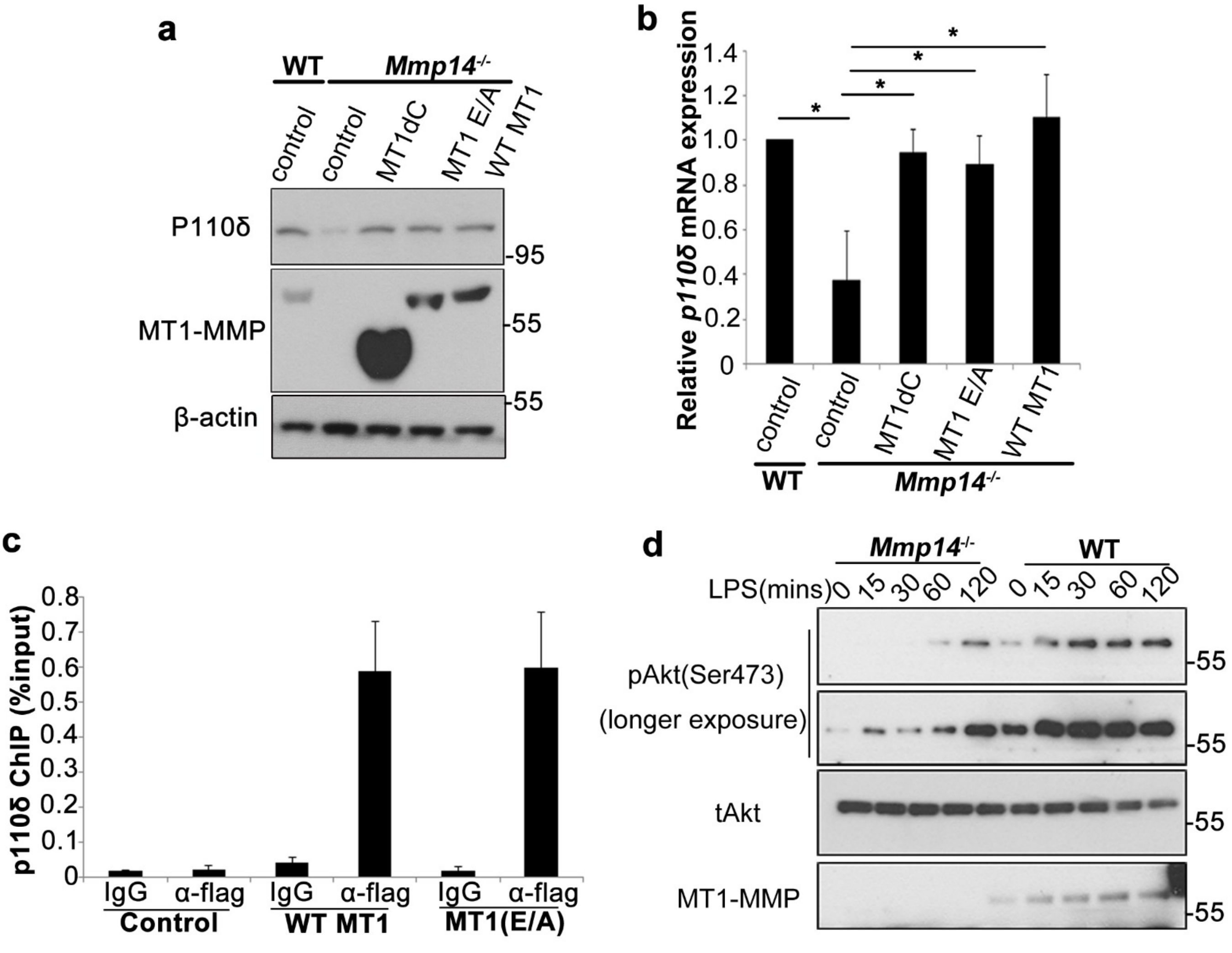

Supplementary Figure 13. Defective PI3Kδ/Akt signaling in *Mmp14*$^{-/-}$ BMM

(a)-(b) Western Blotting **(a)** and qPCR analyses **()** of p110δ in BMMs from WT mice and *Mmp14*$^{-/-}$ mice expressing a control vector, full-length MT1-MMP, the cytosolic domain deleted or the E/A240 MT1-MMP mutant. (*p<0.05, n=3, two-tailed t-test)

(c) Binding of flag-tagged MT1-MMP at the promoter of *p110δ* in *Mmp14*$^{-/-}$ BMMs expressing a control vector, flag-tagged full-length MT1-MMP or flag-tagged mutant MT1-MMP (E/A240), examined by qPCR analyses of chromatin immunoprecipitation (ChIP) assays using flag antibodies (n=3)

(d) Western blotting of phosphorylation of Akt (pAkt) in BMMs from WT mice and *Mmp14*$^{-/-}$ mice upon LPS (1μg/mL) stimulation. Total Akt (tAkt) served as a loading control. Data represent the average ± SEM. The experiments were repeated at least three times.

Supplementary Figure 14

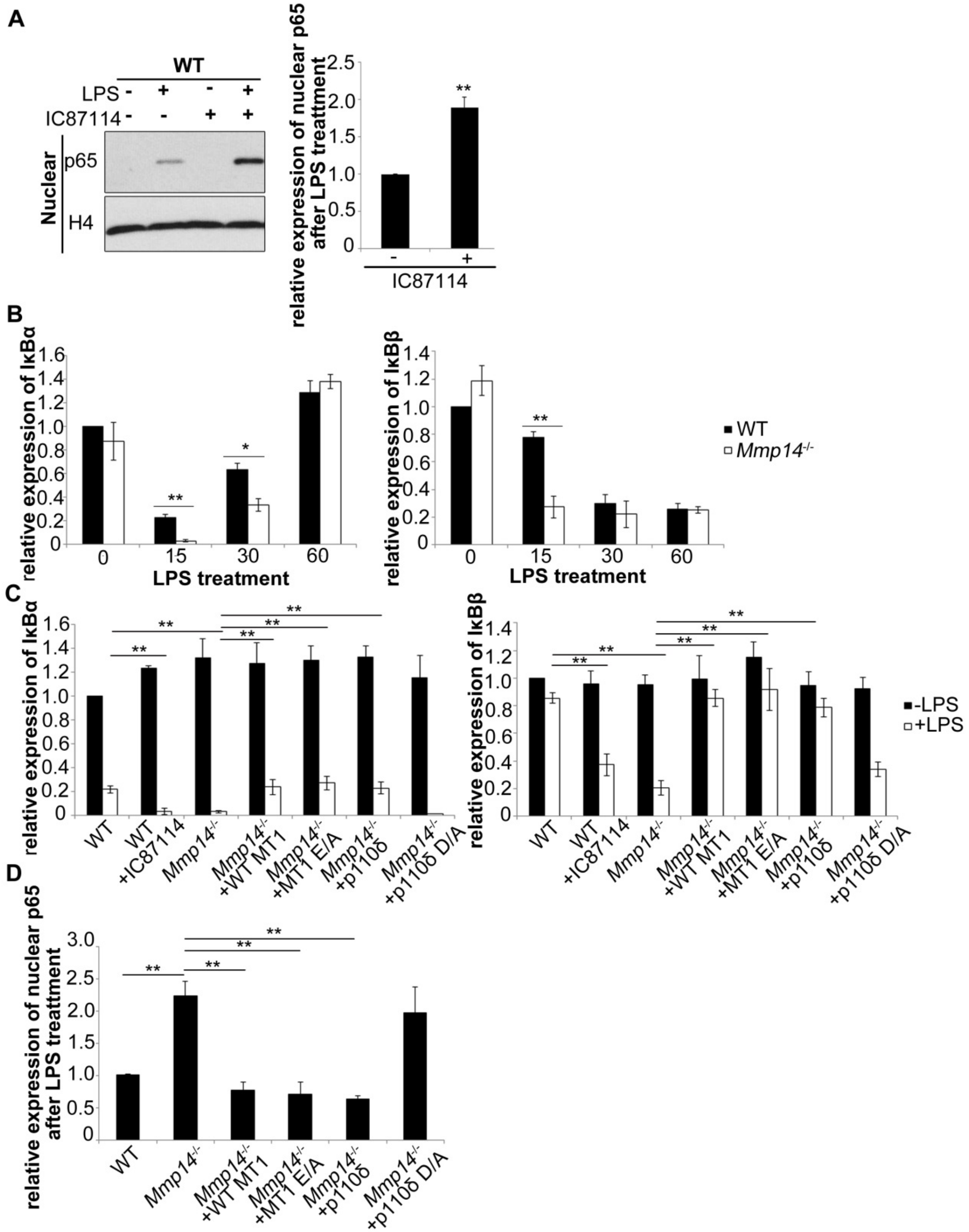

Supplementary Figure 14. Augmented NF-κB signaling in *Mmp14*$^{-/-}$ BMM

(a) Western blotting analyses of p65 expression in the nuclear fractions of *Mmp14*$^{+/+}$ BMMs treated with or without IC87114 after LPS-stimulation. Quantification of nuclear p65 upon LPS stimulation was shown in the right panel (*$p<0.05$;**$p<0.01$, n=3, two-tailed t-test).

(b) Quantification of IκBα and IκBβ expression in relative to β-actin shown in **Fig. 7g**

(*p<0.05,**p<0.01, n=3, two-tailed *t*-test)**.**

(c) Quantification of IκBα and IκBβ expression in relative to β-actin shown in **Fig. 7h** (**p<0.01, n=3, two-tailed *t*-test)**.**

(d) Quantification of nuclear p65 upon LPS stimulation shown in **Fig. 7i** (**p<0.01, n=3, two-tailed *t*-test). Data represent the average ± SEM. The experiments were repeated at least three times.

Supplementary Figure 15

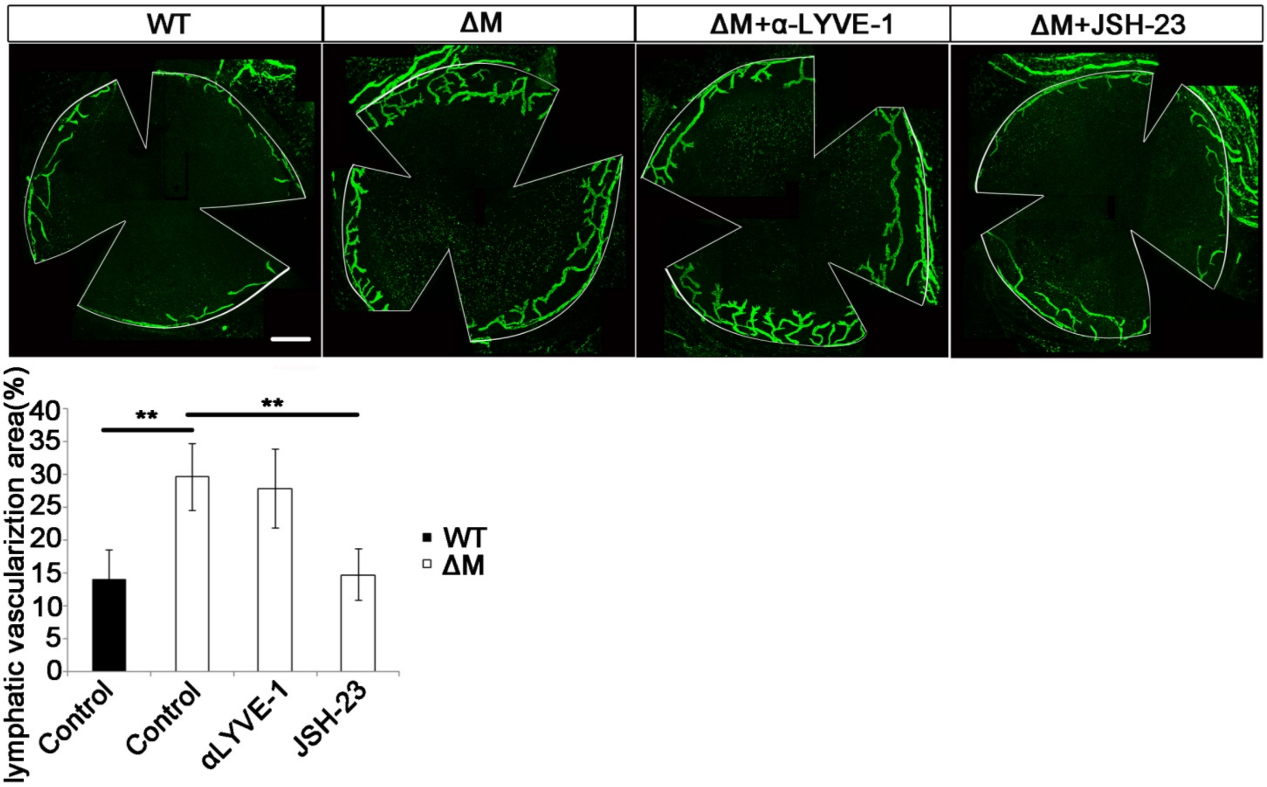

Supplementary Figure 15 Pharmacological inhibition of NF-κB suppresses spontaneous corneal lymphangiogenesis in *Mmp14*$^{flox/flox}$ *LysM-cre* (+) mice

Mmp14$^{f/f}$ *lysM*-Cre^{-} (WT) and *Mmp14*$^{f/f}$ *lysM*-Cre^{+} (ΔM) mice were daily treated with either α-LYVE-1 neutralizing antibody (2 mg/kg) or JSH23 (10 mg/kg) starting from P5. Mice treated with DMSO and control IgG served as controls. Mice were sacrificed at P20 and examined for lymphatic vascularization by whole-mounted staining of corneas with specific antibodies against LYVE-1. The lymphatic vascularization areas of stained corneas were quantified in the lower panel. (**p<0.01; n=4). The statistical analyses were performed by ANOVA, followed by two-tailed, unpaired Student's t test. Data represent the average ± SEM. The experiments were repeated at least three times. Scale bars: 200μm

Supplementary Figure 16

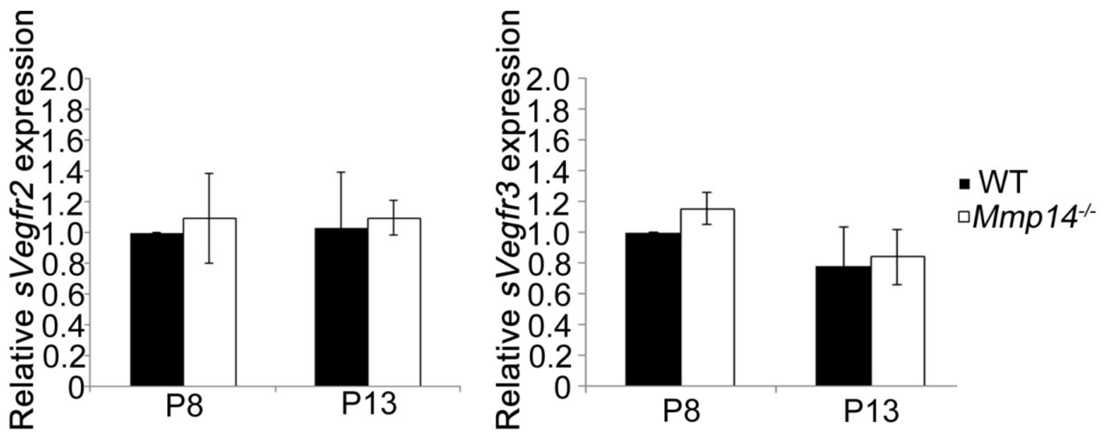

Supplementary Figure 16. Expression of soluble VEGFR2 in *Mmp14*$^{-/-}$ corneas

The expressions of *sVegfr2* (left) and *sVegfr3* (right) in corneas from WT mice and *Mmp14*$^{-/-}$ mice at different developmental stages were examined by qPCR. (n=4) Data represent the average ± SEM. The experiments were repeated at least three times.

Supplementary Figure 17

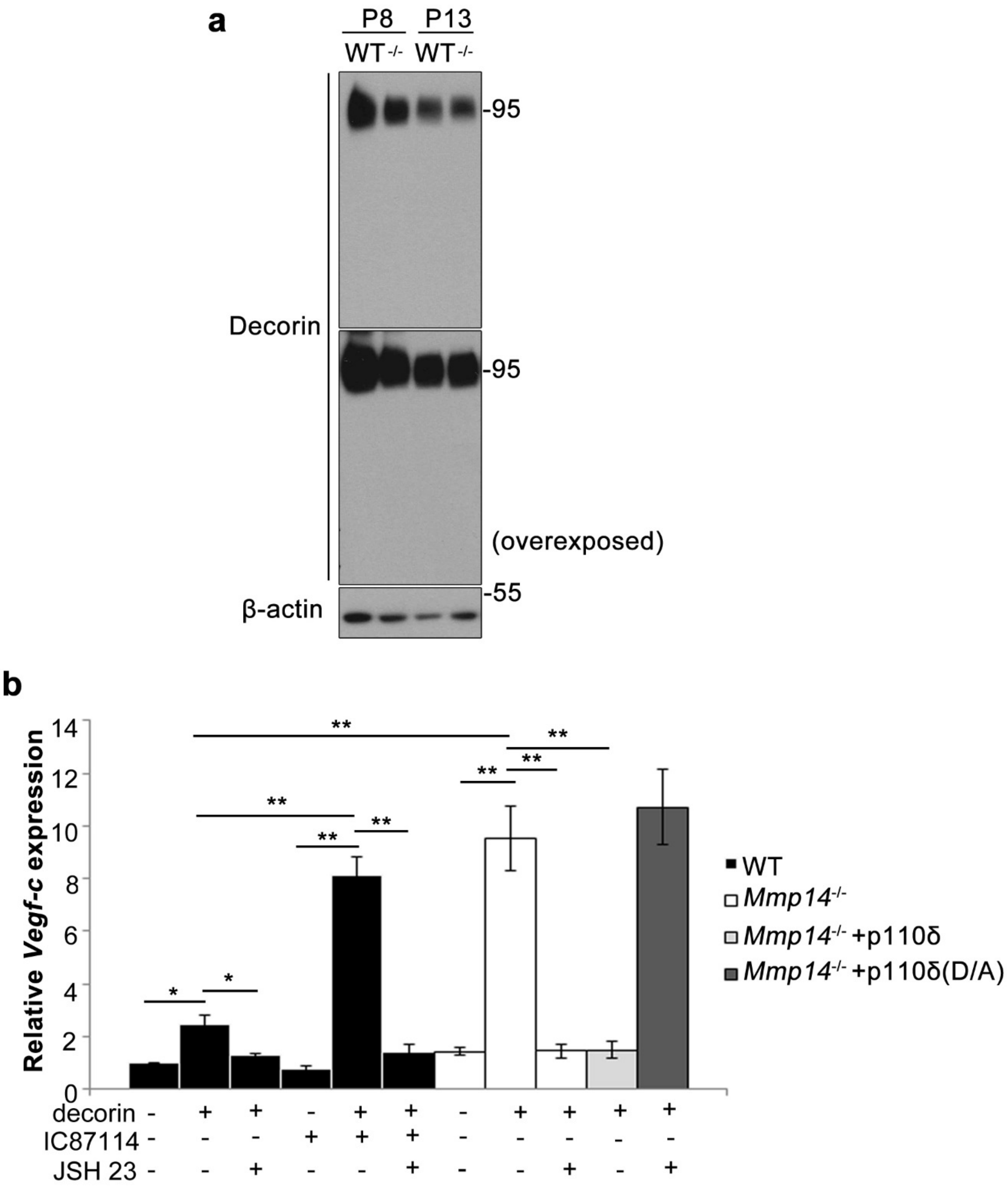

Supplementary Figure 17. Increased production of VEGF-C in response to decorin in *Mmp14*$^{-/-}$ macrophages

(a) The expression of decorin in *Mmp14*$^{-/-}$ corneas is comparable to that in WT corneas. Decorin was detected by Western Blotting using specific antibody against murine decorin. No cleaved fragment of Decorin was detected even in the over exposed film.

(b) qPCR analyses of *Vegf-c* expression in WT BMMs, *Mmp14*$^{-/-}$ BMMs and *Mmp14*$^{-/-}$ BMMs expressing a control vector, full-length p110δ or catalytic inactive p110δ (D/A) stimulated with recombinant decorin (10ng/ml) for 4 hours and assessed for by (*p<0.05;**p<0.01, n=3, two-tailed t-test). Data represent the average ± SEM. The experiments were repeated at least three times.

Supplementary Figure 18

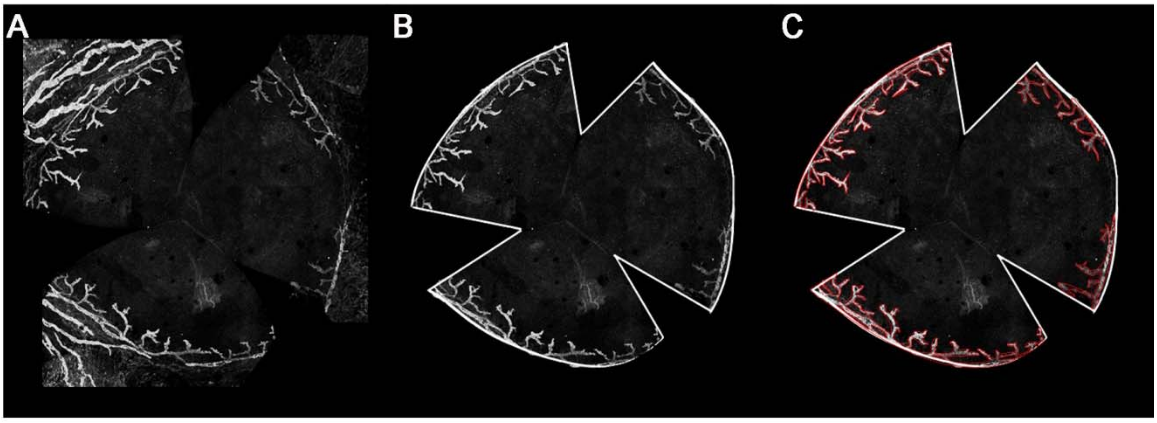

Supplementary Figure 18 Illustration of lymphatic vascularization in corneas

Whole mounted corneas were immunostained by specific antibodies against LYVE-1. The representative images of cornea were adopted from **Fig. 7a**. Images with low magnification (X50) from different areas of corneas were merged to obtain the image of entire corneas **(A)**. The total corneal area was outlined within the innermost lymphatic vessels of the limbus (white lines) **(B)**. The vascularization area was defined by the area of corneas covered with ingrowth lymphatic vessels (red line) **(C)**.

Supplementary Figure 19

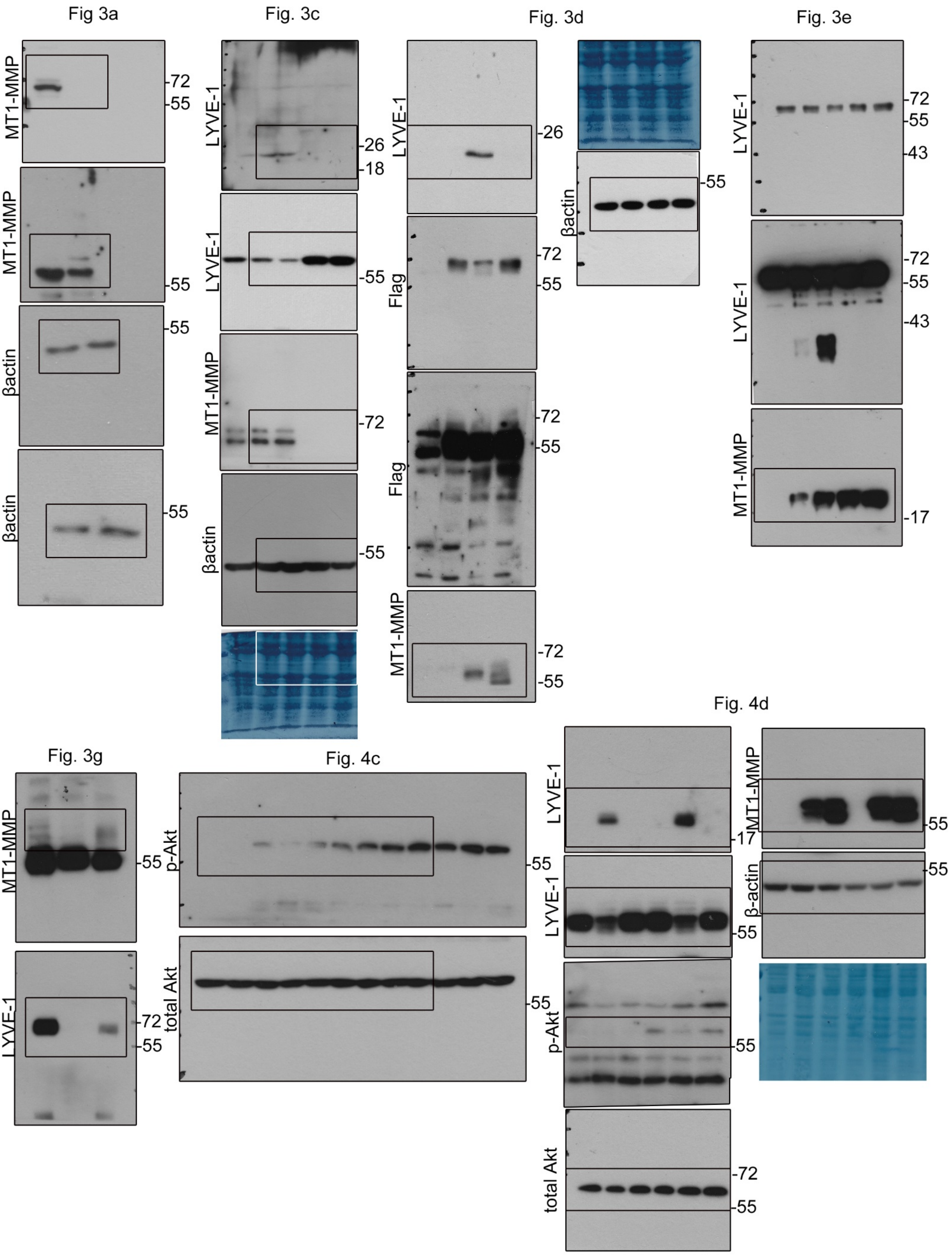

Supplementary Figure 19 The original immunoblots for all main figures and supplementary figures. Black boxed area represents where a panel in each immunoblot was selected for display.

Supplementary Figure 19 (Continued)

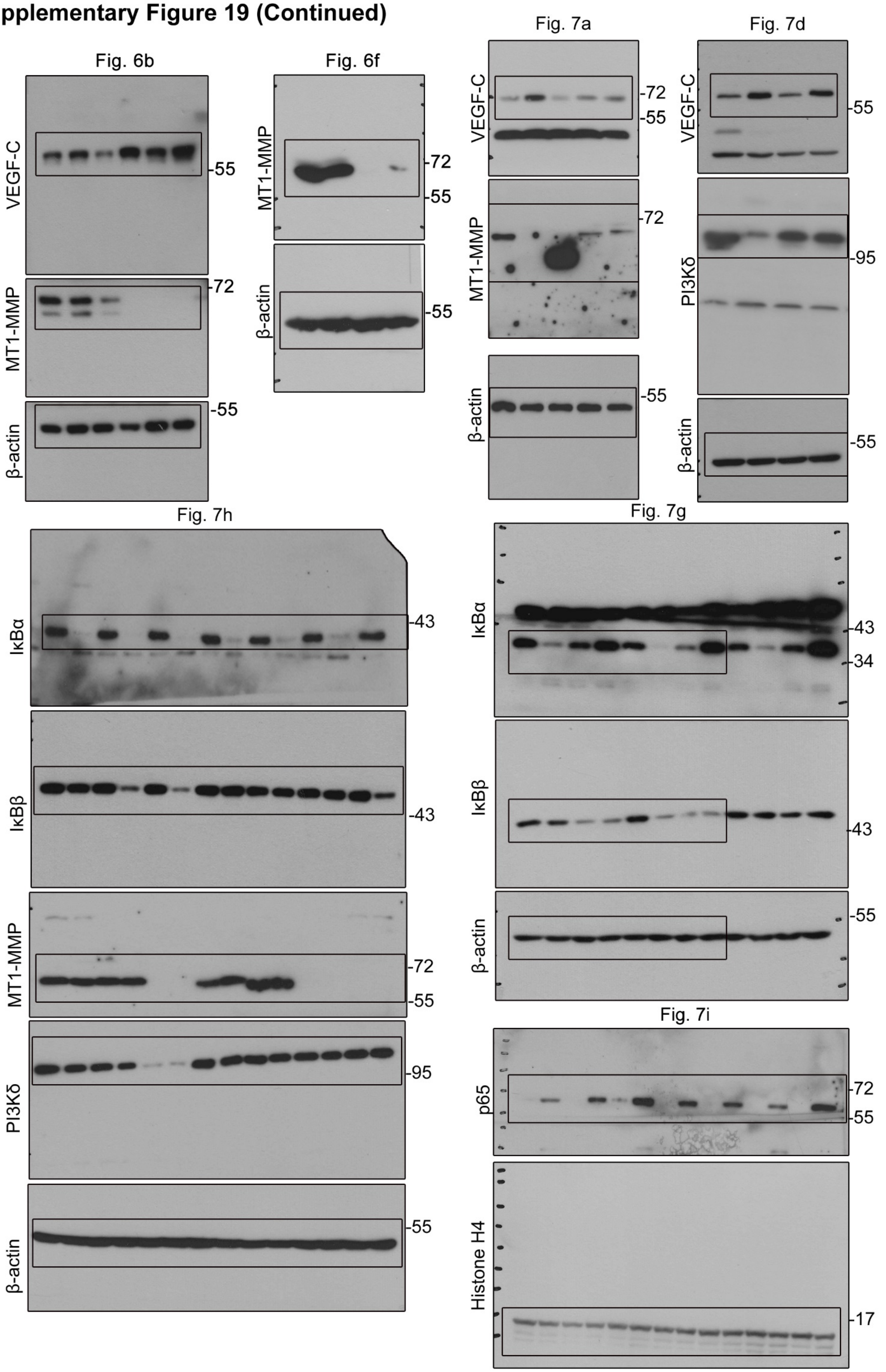

Supplementary Figure 19 (continued)

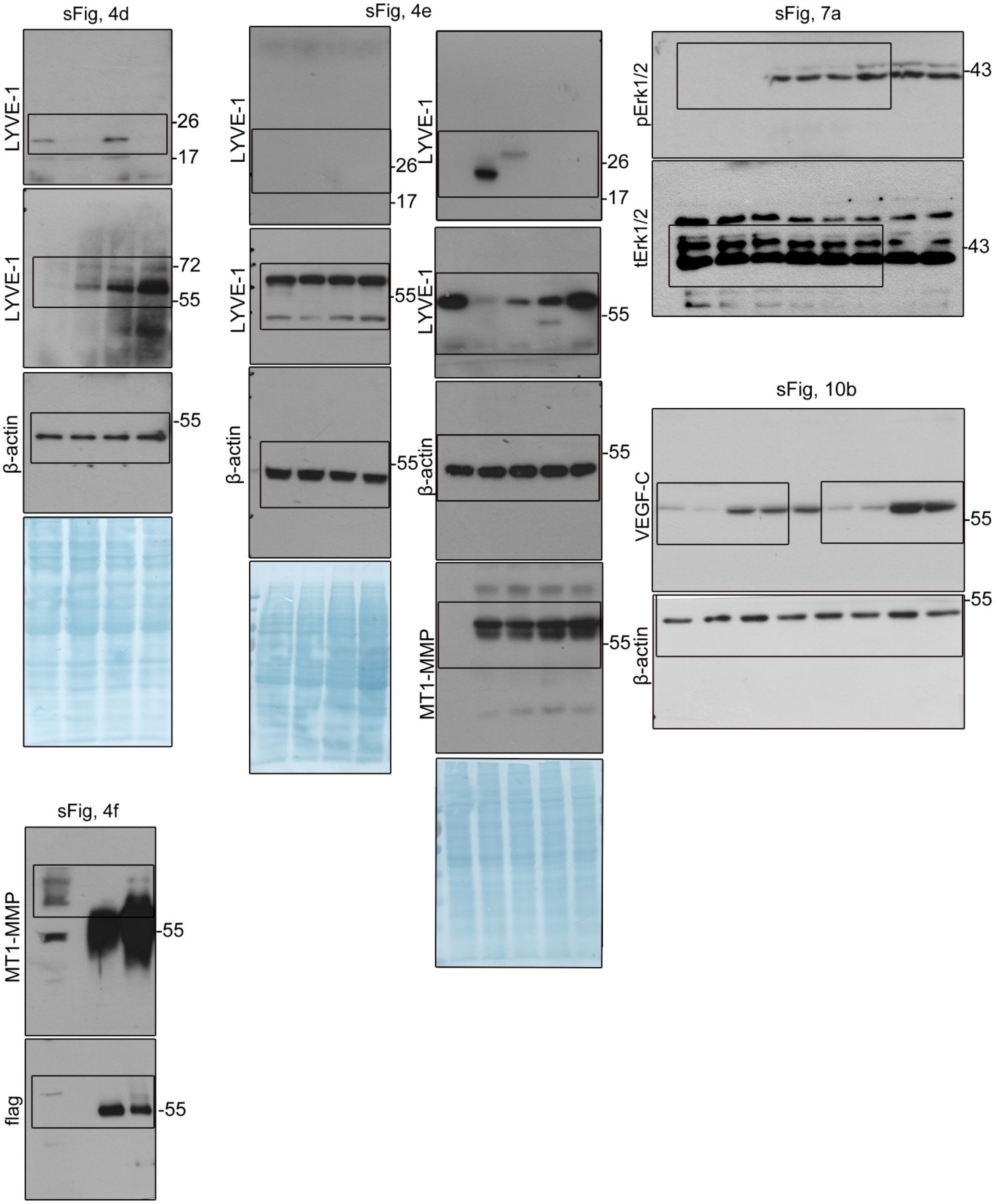

Supplementary Figure 19 (continued)

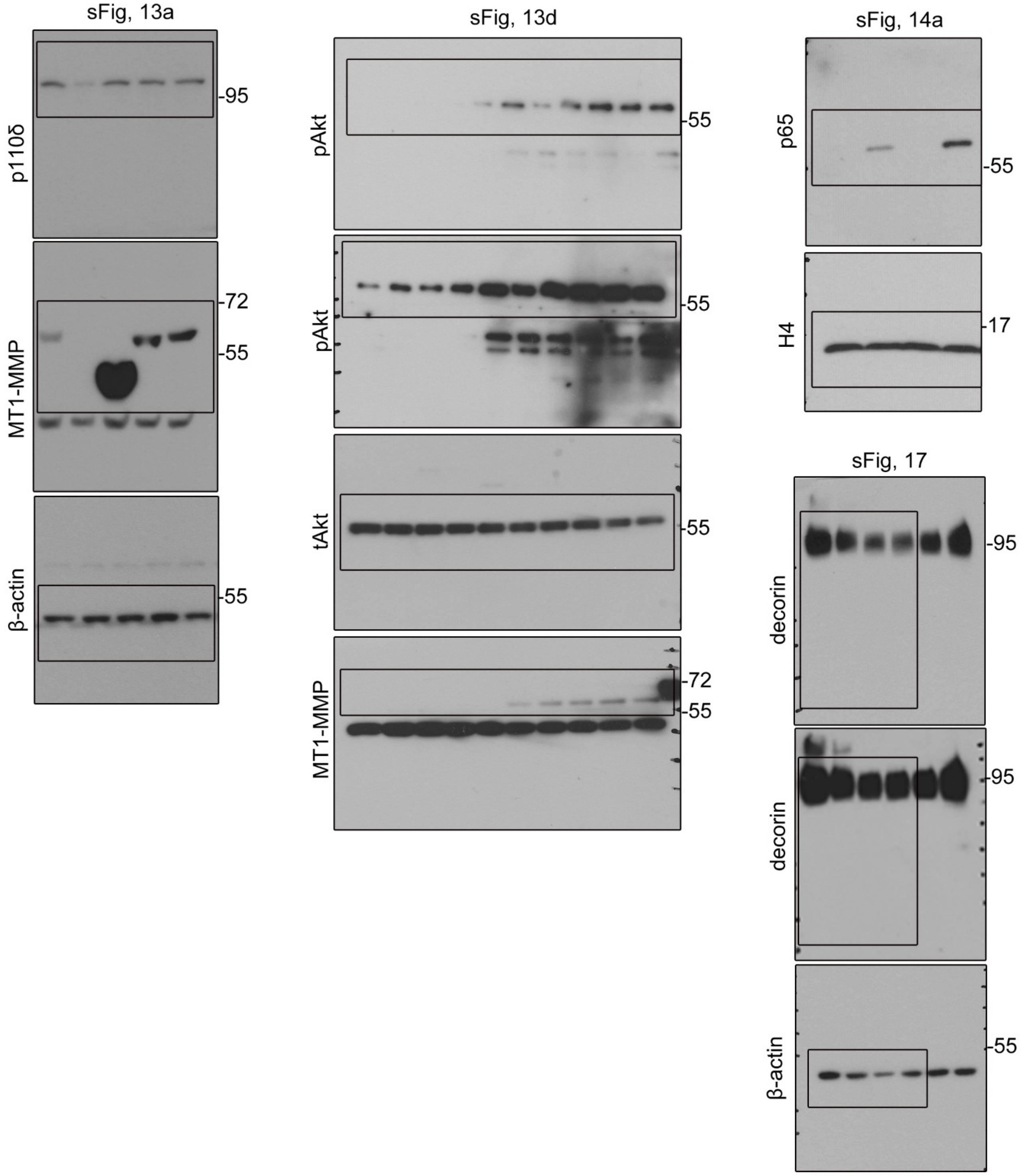

Genes	Forward (5'-3')	Reverse (5'-3')
Vegf-a	CTGCCGTCCGATTGAGA	ACTCCAGGGCTTCATCGTTAC
Vegf-c	CTGGGAAATGTGCCTGTGAATG	ATTCGCACACGGTCTTCTGTAAC
Vegf-d	CAAGACGAGACTCCACTGCC	GCACTCACAGCGATCTTCATC
Il-1β	TTGACGGACCCCAAAAGATGAAGGG	TCCACAGCCACAATGAGTGATACTG
Il-6	AGTCAATTCCAGAAACCGCTATGA	TAGGGAAGGCCGTGGTTGT
Tnf	GGCCTCCCTCTCATCAGTTCTATG	GTTTGCTACGACGTGGGCTACA
Mip-2	TACTGAACAAAGGCAAGGCTAACT	CGAGGCACATCAGGTACGA
Mcp-1	ACTGCATCTGCCCTAAGGTCTT	GCTTCAGATTACGGGTCAACTTC
sVegfr-2	CACCAGTTTGCAAGAACTTGGATGCT	AATTCTGTCACCCAGGGATG
Gapdh	GAGCCAAA AGGGTCATC	GTGGTCATGAGTCCTTC
Vegf-c for ChIP assay	GAGGGCAAAAGTTGCGAGC	GTGAGGCTGAGGTCCTCTCCT
p110δ for ChIP assay	TCCTCTTTGCTTTCTTTCAGAC	CCTCTGCCTGGGAGATTAAA

Supplementary Table 1. Primer sequences used for qRT-PCR
